# ﻿The genus *Batophila* Foudras, 1860 (Coleoptera, Chrysomeliae, Galerucinae, Alticitae) in Taiwan, with descriptions of 11 new species

**DOI:** 10.3897/zookeys.1258.163900

**Published:** 2025-11-03

**Authors:** Chi-Feng Lee

**Affiliations:** 1 Applied Zoology Division, Taiwan Agricultural Research Institute, Taichung 413, Taiwan Applied Zoology Division, Taiwan Agricultural Research Institute Taichung Taiwan

**Keywords:** Host plant, leaf beetles, Melastomataceae, Polygonaceae, Rosaceae, *
Rubus
*, taxonomy

## Abstract

The Taiwanese species of the genus *Batophila* Foudras, 1860 are revised. *Batophila acutangula* Heikertinger, 1921 is removed from the list of Taiwanese fauna. *Batophila taiwanica* Döberl, 2010 is recognized and redescribed. Additionally, eleven new species from Taiwan are described: *B. alishanensis***sp. nov.**, *B. choui***sp. nov.**, *B. chungi***sp. nov.**, *B. houjayi***sp. nov.**, *B. huangi***sp. nov.**, *B. jungchani***sp. nov.**, *B. meihuai***sp. nov.**, *B. tsoui***sp. nov.**, *B. wusheensis***sp. nov.**, *B. yehi***sp. nov.**, and *B. yuae***sp. nov.** The species descriptions include illustrations of aedeagi, antennae, gonocoxae, abdominal ventrite VIII, and spermathecae.

## ﻿Introduction

The flea beetle genus *Batophila* Foudras, 1860 can be recognized easily by the combination of the following characters: general color metallic; anterior coxal cavities open posteriorly; dorsum not pubescent; pronotum evenly convex, without a distinct antebasal, transverse impression; elytron with reduced humeral calli, punctures arranged into 10 rows; hind wings absent; prosternum exceeding posterior margin of coxae; hind tibia with apical, simple spine; tarsomere III bilobed (Yang et al. 2015).

This genus contains 30 species recorded from Oriental and Palearctic regions (Nadein 2013), 24 of them from the Palearctic (Bezděk and Konstantinov 2024). Chûjô (1937) initially recorded the genus from Taiwan based on *B. yangweii* Chen, 1933, which was synonymized with *B. acutangula* Heikertinger, 1921 by Heikertinger (1948). Döberl (2010) added the second species, *B. taiwanica* Döberl, 2010, to the Taiwanese fauna. Adults of the genus are common in various forest types and easily collected by sweeping. More than 3,400 specimens were available for study, including historical collections at several museums, and extensive collections made by the Taiwan Chrysomelid Research Team (TCRT) (Lee 2025). More than 3,000 specimens were collected and deposited at the Taiwan Agricultural Research Institute during 1979–1984. The collecting sites include a number of places along Provincial Highway 14A, including Wushe (霧社, 1250 m a.s.l.), Yu-shih (幼獅, 1750 m a.s.l.), Sungkang (松崗, 2100 m), Meifeng (梅峰, 2150 m a.s.l.), Tsuifeng (翠峰, 2300 m a.s.l.), and Tayuling (大禹嶺, 2560 m a.s.l.). Other sites include Alishan (阿里山, 2400 m a.s.l.), Tungpu (東埔, 1200 m a.s.l.), and Wufeng (五峰, 400 m a.s.l.). The true species diversity and distributions can now be presented based on sufficient material.

## ﻿Materials and methods

For taxonomic study, abdomens of adults were separated from the forebodies and boiled in 10% KOH solution, followed by washing in distilled water to prepare genitalia for illustrations. The genitalia were then dissected from the abdomens, mounted on slides in glycerin, and studied and drawn using a Leica M165 stereomicroscope. For detailed examinations, a Nikon ECLIPSE 50i microscope was used.

At least three males and females from each species were examined to delimit variability of diagnostic characters. For species collected from more than one locality or with color variations, at least one pair of each sex from each locality and color morph was examined. Length was measured from the anterior margin of the eye to the elytral apex, and width at the greatest width of the elytra. Nomenclature for morphological structures of adults follows Duckett and Daza (2004). Names of plant species follow the Taiwan Encyclopedia of Life (2024).

Specimens studied herein are deposited at the following institutes and collections:

**KMNH**Kitakyushu Museum of Natural History and Human History, Kitakyushu, Japan [Yûsuke Minoshima]

**MHNG**Muséum d’Histoire Naturelle, Genève, Switzerland [Giulio Cuccodoro]

**NHMB**Naturhistorisches Museum, Basel, Switzerland [Matthias Borer]

**NHMUK**The Natural History Museum, London, UK [Michael F. Geiser]

**NMNS**National Museum of Natural Science, Taichung, Taiwan [Bao-Cheng Lai]

**TARI**Applied Zoology Division, Taiwan Agricultural Research Insitute, Taichung, Taiwan [Chi-Feng Lee]

Exact label data are cited for all type specimens of described species; a double slash (//) divides the data on different labels and a single slash (/) divides the data in different rows. Other comments and remarks are in square brackets: [p] – preceding data are printed, [h] – preceding data are handwritten, [r] – red label, [w] – white label.

## ﻿Taxonomic account

### ﻿ Batophila acutangula

Taxon classificationAnimaliaColeopteraChrysomelidae

Heikertinger, 1921

FFEDA544-141B-576D-85AE-D2606D5EB2D6

Fig. 1A–C

Batophila acutangula Heikertinger, 1921: 7.
Batophila yangweii Chen, 1933: 250; Heikertinger, 1948: 53 (as synonym for B. acutangula).

#### Type specimens examined.

***Lectotype*** ♂ (NHMB): “H. Frieb / Perewaja-Rjetsch / Ka-Tal, 7–8 km / nördlich von / Wladiwostok [h, w] // Batophila / acutangula m. [h] / det. Heiktgr. [p, w] // Batoph. / acutang. / Type! [h, r] // 1953 Coll. / Heikertinger [p, w] // lectotype [h] / J. Bechyne det., 1956 [p, w]”

#### Notes.

*Batophila acutangula* Heikertinger, 1921 is not found in Taiwan although it was recorded from Taiwan previously (Chûjô 1937). Voucher specimens of *B. yangweii* (Chûjô 1937) are identified as *B. houjayi* sp. nov., *B. huangi* sp. nov., *B. taiwanica* Döberl, 2010, *B. tsoui* sp. nov., *B. wusheensis* sp. nov., and *B. yuae* sp. nov. Voucher specimens of *B. acutangula* (Kimoto 1971, 1989) are identified as *B. alishanensis* sp. nov., *B. choui* sp. nov., *B. houjayi* sp. nov., *B. taiwanica* Döberl, 2010. Several subspecies of *B. acutangula* in Japan were synonymized by Kimoto (1966); however, they may represent valid species since their aedeagi are different in shape, as illustrated by Kimoto (1966).

**Figure 1.**
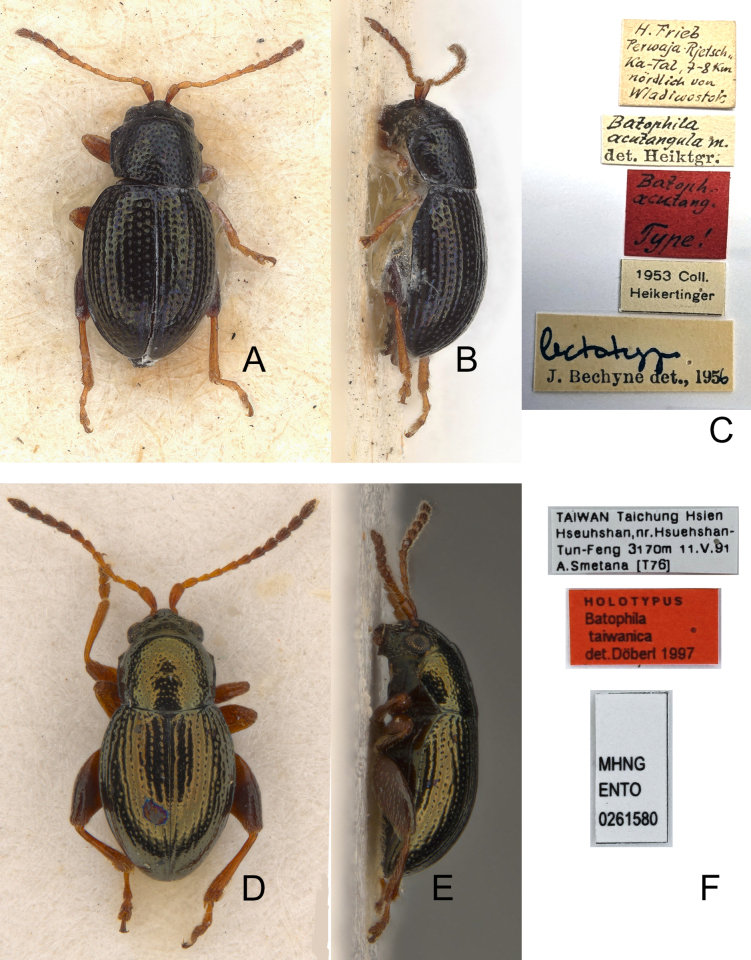
Type specimens and labels A. *Batophila acutangula* Heikertinger, 1921, lectotype, dorsal view; B. Ditto, lateral view; C. Labels pinned with lectotype; D. *B. taiwanica* Döberl, 2010, holotype, dorsal view; E. Ditto, lateral view; F. Labels pinned with holotype.

### ﻿ Batophila alishanensis sp. nov.

Taxon classificationAnimaliaColeopteraChrysomelidae

5DB927FF-CBAD-5AEB-A8FB-5CDE62EEEBAF

https://zoobank.org/54992EF9-7381-4B29-91DD-F31FEE2343D3

Figs 2, 3, 4

Batophila acutangula : Kimoto 1989: 269 (part).

#### Type specimens examined (n = 47).

***Holotype*** ♂ (TARI): **Taiwan** • **Chiayi**: Alishan (阿里山), 17–20.VIII.1982, leg. K. C. Chou & C. C. Pan. ***Paratypes*** • 9♂, 8♀ (TARI), same data as holotype; 1♀ (KMNH), same locality, 5.V.1971, leg. K. Kamiya; • 1♂, 1♀ (KMNH), same but with “25.V.1971"; 1♂, 2♀♀ (KMNH), same but with “26.V.1971"; • 2♂♂, 2♀♀ (KMNH), same locality, 22–25.VI.1974, leg. M. Owada; • 4♂♂, 9♀♀ (TARI), same locality, 5–9.VIII.1981, leg. L. Y. Chou & S. C. Lin; • 1♂, 1♀ (KMNH), same locality, 7.IX.1986, leg. K. Baba, identified as *B. acutangula* by Kimoto (1989); • 1♂ (KMNH), same locality, 6.VIII.1990, leg. S. Kimoto; • **Chiayi**: 1♂, 3♀♀ (NMNS), Lulinshan (鹿林山), 18.V.1991, leg. C. C. Chiang; • **Nantou**: 1♀ (TARI), Niitakayama (= Yushan, 玉山), 17.VII.1941, leg. S. Miyamoto; • 1♀ (TARI), Tatachia (塔塔加), 16–23.VI.2007, leg. C.-S. Tung.

#### Diagnosis.

**Figure 2.**
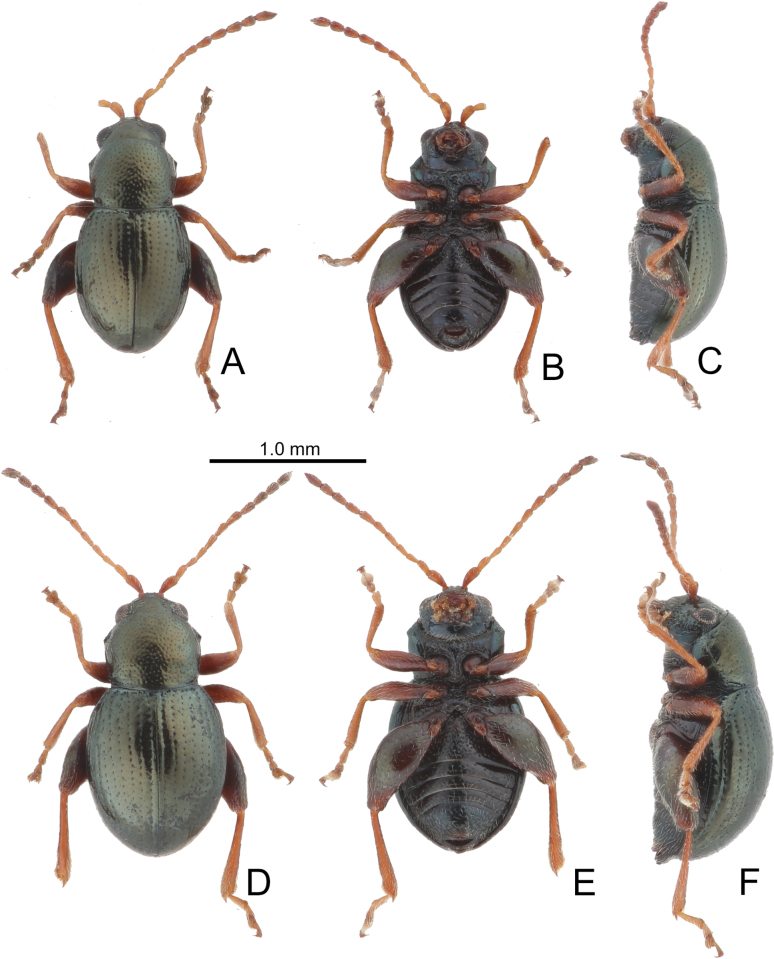
Habitus of *Batophila alishanensis* sp. nov. A. Male, paratype, dorsal view; B. Ditto, ventral view; C. Ditto, lateral view; D. Female, paratype, dorsal view; E. Ditto, ventral view; F. Ditto, lateral view.

**Figure 3.**
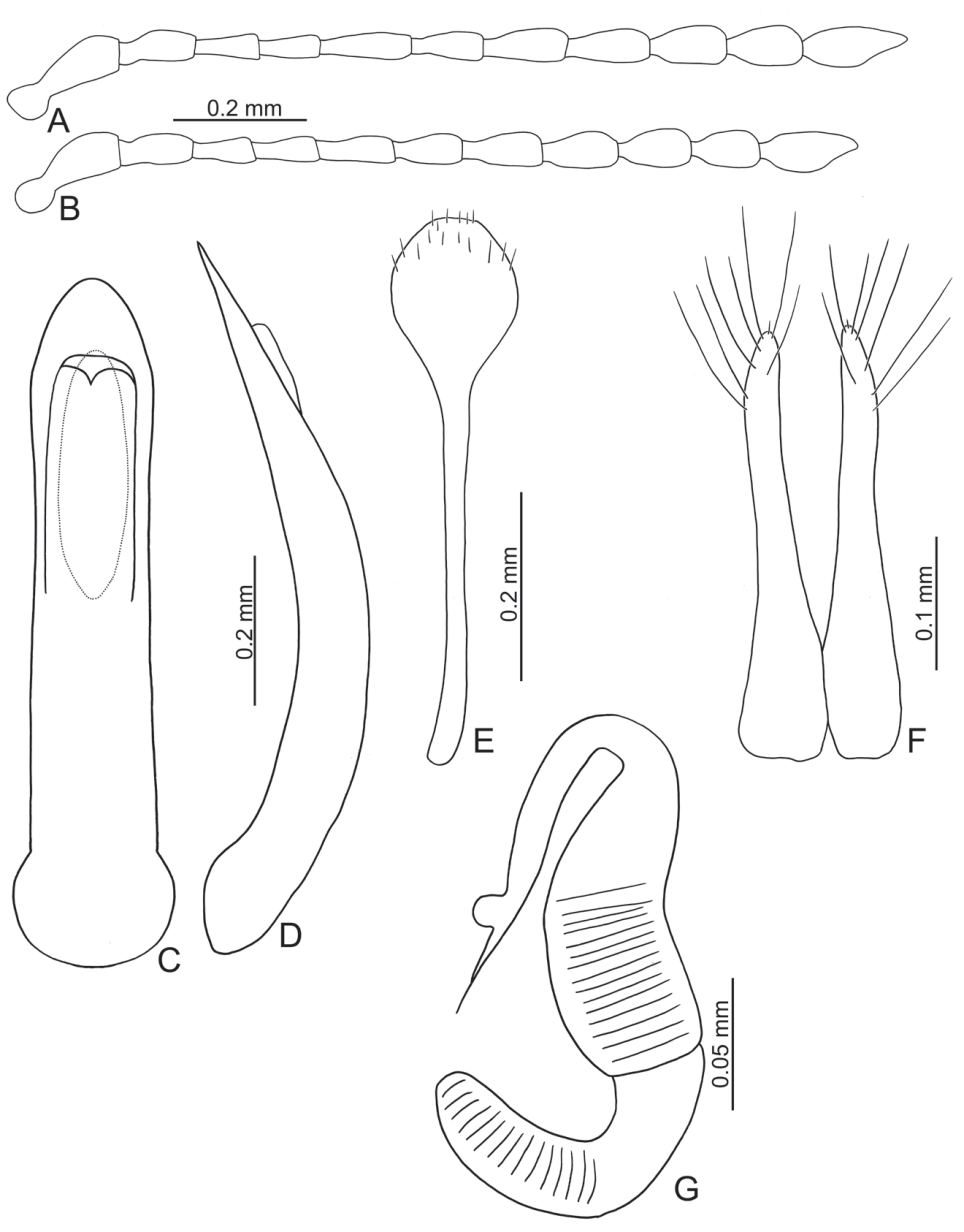
*Batophila alishanensis* sp. nov. A. Antenna, male; B. Antenna, female; C. Aedeagus, dorsal view; D. Aedeagus, lateral view; E. Abdominal ventrite VIII, female; F. Spermatheca; G. Gonocoxae.

Adults of *B. alishanensis* sp. nov. are similar to those of *B. wusheensis* sp. nov., *B. houjayi* sp. nov. (Figs 10, 12), *B. yuae* sp. nov., *B. jungchani* sp. nov., and *B. huangi* sp. nov. (Fig. 16) in possessing truncate elytral apices. It can be recognized easily by fine punctures on elytra (Fig. 2). The aedeagus of this new species is similar to that of the sympatric species *B. houjayi* sp. nov. but differs by the wider subapical area (Fig. 3C) [apically narrow aedeagus in *B. houjayi* sp. nov. (Fig. 11C)].

#### Description.

Male. Length 1.44–1.61 mm, width 0.74–0.80 mm. General color metallic dark bronze (Fig. 2A–C); legs yellowish but hind femora darkened. Antenna (Fig. 3A) filiform and antennomeres VIII–X wide, ratio of length of antennomeres I–XI to length of antennomere I 1.0: 0.6: 0.5: 0.5: 0.8: 0.6: 0.7: 0.7: 0.6: 0.6: 0.9; ratio of length to width of antennomeres I–XI 2.9: 2.5: 2.3: 2.5: 3.4: 2.5: 2.4: 2.4: 1.9: 1.9: 2.6. Pronotum 1.19–1.25× wider than long; lateral margins slightly rounded, anterolateral angles separated from lateral margins by weak emarginations, slightly narrowed basally, distance between anterolateral angles 1.13–1.17× wider than basal margin. Elytra 1.27–1.30× longer than wide; lateral margins rounded, widest at basal 1/5, apex truncate; dorsoventrally flattened, apex visible in dorsal view; disc with longitudinal lines of fine punctures and with indistinct longitudinal grooves along lines present near base and sides. Tarsomeres I of front and middle legs slightly swollen. Aedeagus (Fig. 3C, D) elongate, 5.8× longer than wide; narrowest at apical 1/3, apically widened towards apical 1/7 and then narrowed, apex widely rounded, basally widened near base; dorsal opening starting from apical 1/10 and basally membranous, tectum composed of two lobes, mostly membranous; slightly curved in lateral view; ventral surface with membranous area narrower than dorsal opening, starting from apical 1/10 to 2/5.

Female (Fig. 2D–F). Length 1.75–1.93 mm, width 0.85–0.96 mm. Antennae similar to males, ratio of length of antennomeres I–XI to length of antennomere I (Fig. 3B) 1.0: 0.6: 0.5: 0.5: 0.7: 0.6: 0.7: 0.7: 0.6: 0.6: 0.9; ratio of length to width of antennomere I–XI 2.6: 2.3: 2.4: 2.3: 2.8: 2.2: 2.2: 2.1: 1.8: 1.9: 2.5. Elytra 1.37–1.38× longer than wide; lateral margins rounded, widest at basal 1/3, apex truncate; dorsoventrally convex, apex not visible in dorsal view; disc with longitudinal lines of fine punctures and with indistinct longitudinal grooves along lines, reduced in some individuals. Gonocoxae (Fig. 3F) slender, connected with each other at basal 1/5; each gonocoxa with seven long setae and one tiny seta from apical 1/5 to apex, subapically slightly curved. Ventrite VIII (Fig. 3E) weakly sclerotized apically, with several short setae at apical area, and some tiny setae at apical margin, spiculum extremely elongate. Spermathecal receptaculum (Fig. 3G) strongly swollen, with transverse wrinkles at basal 1/2; pump wide and curved, with transverse wrinkles at apical 2/3; sclerotized spermathecal canal moderately long before base of spermathecal gland.

#### Food plants.

Rosaceae: *Rubus* sp.

#### Etymology.

This new species is named after its type locality, Alishan (阿里山).

#### Distribution.

**Figure 4.**
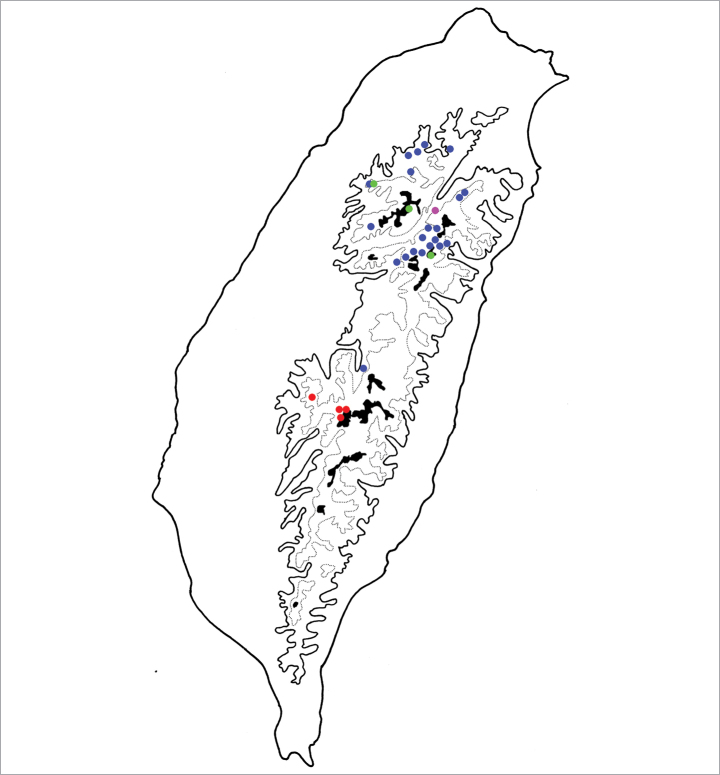
Distribution map of *Batophila* species, solid line: 1000 m a.s.l., broken line: 2000 m a.s.l., black areas: 3000 m a.s.l. Red dots *B. alishanensis* sp. nov. Purple dots *B. taiwanica* Döberl Green dots *B. yehi* sp. nov. Brown dots *B. meihuai* sp. nov.

Only known from the abovementioned localities, which are alpine habitats in southern Taiwan (Fig. 4).

### ﻿ Batophila choui sp. nov.

Taxon classificationAnimaliaColeopteraChrysomelidae

B2C97F06-5801-5200-BB98-28B3C699CEA7

https://zoobank.org/96FCCE48-AD8C-4E36-BFA8-14DCAD085CE2

Figs 5, 6, 7

Batophila acutangula : Kimoto 1971: 269 (part).

#### Type specimens examined (n = 558).

***Holotype*** ♂ (TARI). **Taiwan** • **Nantou**: Meifeng (梅峰), 7–9.V.1981, leg. K. S. Lin & S. C. Lin. ***Paratypes*** • **Hualien**: 1♂, 1♀ (TARI), Tayuling (大禹嶺), 9–16.VI.1980, leg. K. S. Lin & B. H. Chen; • **Nantou**: 11♂♂, 7♀♀ (TARI), Meifeng (梅峰), 10.V.1979, leg. K. C. Chou; • 1♂, 2♀♀ (TARI), same locality, 18.VII.1979, leg. K. C. Chou; • 6♂♂, 8♀♀ (TARI), same locality, 2–4.VI.1980, leg. L. Y. Chou & C. C. Chen; • 6♂♂, 9♀♀ (TARI), same locality, 8.VI.1980, leg. K. S. Lin & B. H. Chen; • 3♂♂, 9♀♀ (TARI), same locality, 26.VIII.1980, leg. K. S. Lin & C. H. Wang; • 1♂, 1♀ (TARI), same locality, 5–9.X.1980, leg. C. C. Chen & C. C. Chien; • 25♂♂, 18♀♀ (TARI), same locality, 7–9.V.1981, leg. K. S. Lin & S. C. Lin; • 49♂♂, 25♀♀ (TARI), same locality, 24–26.VI.1981, leg. K. S. Lin & W. S. Tang; • 6♂♂, 6♀♀ (TARI), same locality, 28–29.VIII.1981, leg. L. Y. Chou & S. C. Lin; • 6♂♂, 1♀ (TARI), same locality, 22.V.1982, leg. L. Y. Chou; • 33♂♂, 22♀♀ (TARI), same locality, 15.VII.1982, S. C. Lin & C. N. Lin; • 9♂♂, 13♀♀ (TARI), same locality, 31.VIII.–2.IX.1982, leg. L. Y. Chou & K. C. Chou; • 45♂♂, 25♀♀ (TARI), same locality, 4–7.X.1982, leg. K. C. Chou; 1♀ (TARI), same locality, 19–21.IV.1983, leg. K. C. Chou & S. P. Huang; • 5♂♂, 1♀ (TARI), same locality, 30.VII.1983, leg. L. Y. Chou; • 1♂, 2♀♀ (TARI), same locality, 8–11.V.1984, leg. K. C. Chou & C. C. Pan; • 1♂ (TARI), same locality, 23.VII.1984, leg. K. S. Lin; • 1♀ (NMNS), same locality, 9.I.–6.II.2007, leg. C. S. Lin & W. T. Yang, Malaise trap; • 2♂♂, 4♀♀ (TARI), same locality, 20.IV.2025, leg. C.-F. Lee; • 17♂♂, 5♀♀ (TARI), Sungkang (松崗), 15–17.VIII.1984, leg. K. C. Chou; • 10♂♂, 5♀♀ (TARI), same locality, 13–15.IX.1984, leg. K. S. Lin & S. C. Lin; • 2♂♂, 2♀♀ (KMNH), same locality, 2.VIII.1990, leg. S. Kimoto, of which one male and one female identified as *B. acutangula* by Kimoto in 1990; • 1♂, 1♀ (TARI), same locality, 20.IV.2011, leg. C.-F. Lee; • 4♂, 2♀ (KMNH), Sungkang (松崗) – Tsifen (sic!) (翠峰), 29.VI.1965, leg. S. Kimoto, identified as *B. acutangula* by Kimoto (1971); • 1♀ (NHMUK), (near Sungkang, 松崗) sheep farm, 24°03.121'N, 121°09.643'E, 1916 m, 7.VIII.2008, leg. M. V. L. Barclay & Mendel; • 4♂♂, 1♀ (TARI), Tsuifeng (翠峰), 21.VI.1979, leg. K. S. Lin & B. H. Chen; • 4♀♀ (TARI), same locality, 3.VI.1980, leg. L. Y. Chou & C. C. Chen; • 45♂♂, 28♀♀ (TARI), same locality, 25–27.VI.1981, leg. K. S. Lin & W. S. Tang; • 10♂♂, 3♀♀ (TARI), same locality, 1–3.VIII.1981, leg. T. Lin & W. S. Tang; • 1♂, 1♀ (TARI), same locality, 27.VIII.1981, leg. L. Y. Chou & S. C. Lin; • 1♂, 1♀ (TARI), same locality, 23.V.1982, leg. L. Y. Chou; • 4♂♂, 4♀♀ (TARI), same locality, 1–3.IX.1982, leg. L. Y. Chou & K. C. Chou; • 3♀♀ (TARI), same locality, 20.IV.1983, leg. K. C. Chou & S. P. Huang; • 1♂ (TARI), same locality, IV.1984, Malaise trap, leg. K. S. Lin & K. C. Chou; • 1♂ (TARI), same but with “V.1984"; • 5♂♂ (TARI), same locality, 5.VIII.1984, leg. K. S. Lin; • 3♂♂ (TARI), same locality, 15–16.VIII.1984, leg. K. C. Chou; • 1♂, 5♀ (TARI), same locality, 12–14.IX.1984, leg. K. S. Lin & S. C. Lin; • 1♀ (TARI), Yu-shih (幼獅), 4.VIII.1981, leg. T. Lin & W. S. Tang; • **Taichung**: 1♂ (TARI), Anmashan (鞍馬山), 6–7.VII.1979, leg. L. Y. Chou; • 1♀ (TARI), same but with “6–9.VII.1979"; • 1♀ (NMNS), same locality, 1.V.1990, leg. C. C. Chiang; • 2♀♀ (TARI), same locality, 21.IV.2010, leg. C.-F. Lee; • 8♀♀ (TARI), Chiapaotai (佳保台)—Liming (黎明), 4.VI.1942, leg. S. Issiki.

#### Diagnosis.

**Figure 5.**
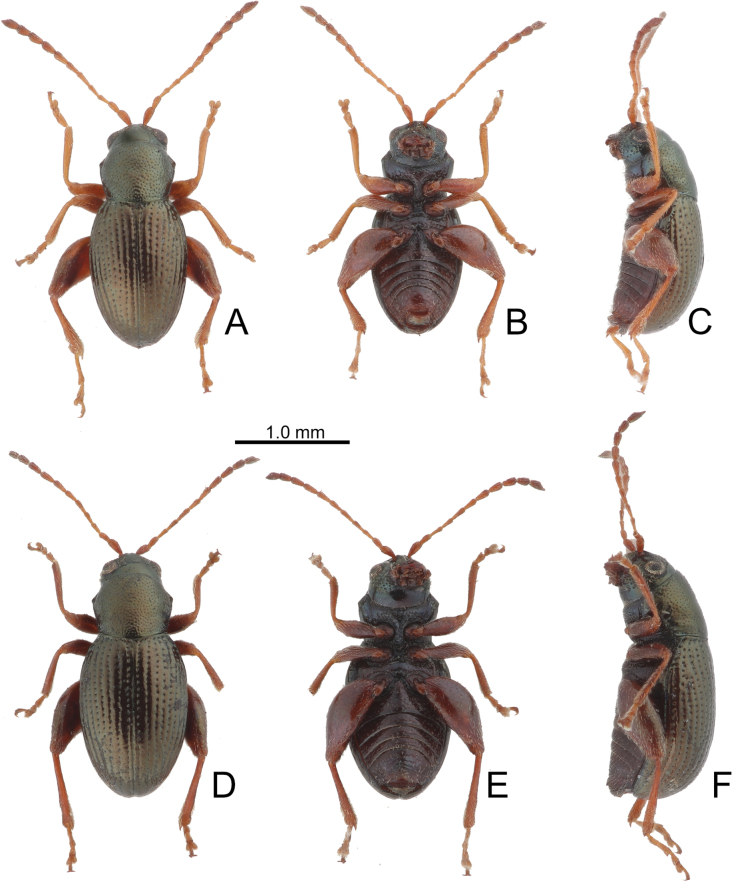
Habitus of *Batophila choui* sp. nov. A. Male, paratype, dorsal view; B. Ditto, ventral view; C. Ditto, lateral view; D. Female, paratype, dorsal view; E. Ditto, ventral view; F. Ditto, lateral view.

**Figure 6.**
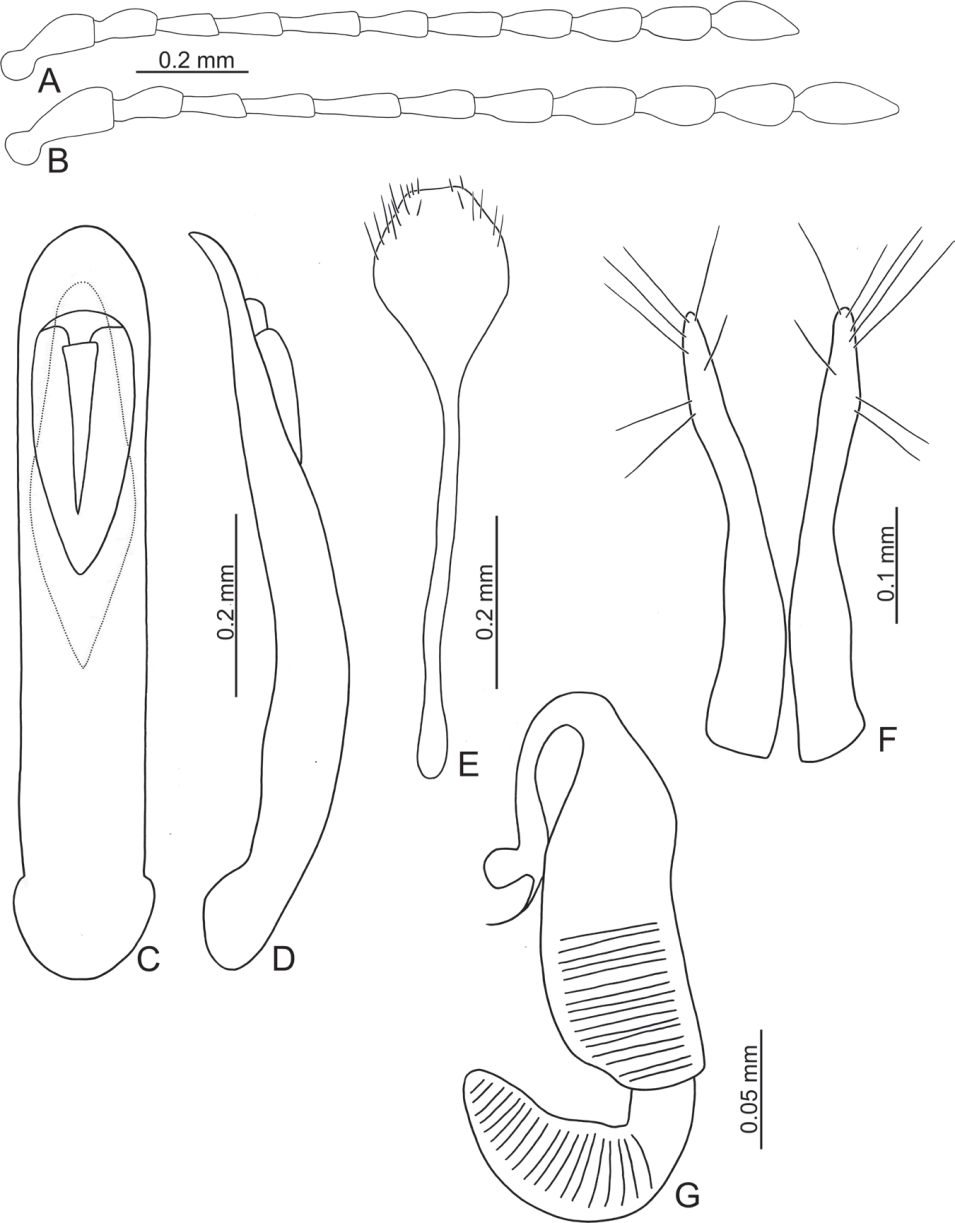
*Batophila choui* sp. nov. A. Antenna, male; B. Antenna, female; C. Aedeagus, dorsal view; D. Aedeagus, lateral view; E. Abdominal ventrite VIII, female; F. Spermatheca; G. Gonocoxae.

Adults of *B. choui* sp. nov., *B. chungi* sp. nov., and *B. tsoui* sp. nov. are recognized by their strongly apically narrowed elytra, and divergent elytral apex. They differ by the presence of convex elytra and elytral apices not visible in dorsal view in both sexes (Fig. 5C, E) [flattened elytra in males but convex elytra in females of *B. chungi* sp. nov. and *B. tsoui* sp. nov. (Fig. 21C, E)], and parallel-sided aedeagus (Fig. 6C) [widened apex of aedeagus in *B. chungi* sp. nov. (Fig. 8C) and *B. tsoui* sp. nov. (Fig. 22C)].

#### Description.

Male. Length 1.99–2.18 mm, width 0.83–0.90 mm. General color metallic dark bronze (Fig. 5A–C); legs yellowish but femora of hind legs darkened. Antenna (Fig. 6A) filiform and antennomeres VIII–X wide, ratio of length of antennomeres I–XI to length of antennomere I 1.0: 0.6: 0.6: 0.6: 0.8: 0.7: 0.7: 0.7: 0.7: 0.7: 0.9; ratio of length to width of antennomeres I–XI 2.8: 2.3: 2.6: 2.9: 3.4: 2.6: 2.8: 2.6: 2.1: 2.1: 2.3. Pronotum 1.13–1.14× wider than long; lateral margins slightly rounded, anterolateral angles separated from lateral margins by weak emargination, slightly and basally narrowed, distance between anterolateral angles 1.11–1.17× wider than basal margin. Elytra 1.52–1.54× longer than wide; lateral margins rounded, widest at basal 1/5, apically and strongly narrowed, apex truncate but diverge; dorsoventrally convex, apex not visible in dorsal view; disc with longitudinal lines of extremely coarse punctures and with distinct longitudinal grooves along punctures, punctures and grooves apically abbreviated from apical 1/3. Tarsomeres I of front and middle legs slightly swollen. Aedeagus (Fig. 6C, D) elongate, 6.0× longer than wide; parallel-sided, apex widely rounded; dorsal opening starting from apical 1/10–1/3, tectum composed of three lobes, median lobe more basal relative to lateral lobes, apical margin truncate, mostly membranous; slightly curved in lateral view, apex moderately curved; ventral surface with membranous area wider than dorsal opening, starting from apical 1/12–1/2.

Female (Fig. 5D–F). Length 2.20–2.38 mm, width 0.96–1.02 mm. Antennae similar to males, ratio of length of antennomeres I–XI to length of antennomere I (Fig. 6B) 1.0: 0.6: 0.6: 0.6: 0.8: 0.7: 0.7: 0.7: 0.7: 0.7: 0.9; ratio of length to width of antennomeres I–XI 2.5: 2.1: 2.7: 3.0: 3.5: 2.7: 2.4: 2.1: 2.0: 1.9: 2.5. Elytra 1.53–1.62× longer than wide; lateral margins rounded, widest at basal 1/5, apically and strongly narrowed, apex truncate but diverge; dorsoventrally convex, apex curved strongly downward in lateral view; disc with longitudinal lines of extremely coarse punctures and with distinct longitudinal grooves along punctures, punctures and grooves apically abbreviated from apical 1/3. Gonocoxae (Fig. 6F) slender, connected at basal 1/5; each gonocoxa with seven long setae and one tiny seta from apical 1/5 to apex, subapically slightly curved. Ventrite VIII (Fig. 6E) weakly sclerotized apically, with several short setae at apical area, and some tiny setae at apical margin, spiculum extremely elongate. Spermathecal receptaculum (Fig. 6G) strongly swollen, with transverse wrinkles at basal 1/2; pump wide and curved, with transverse wrinkles at apical 2/3; sclerotized spermathecal canal moderately long before base of spermathecal gland.

#### Food plants.

Rosaceae: *Rubus corchorifolius* L. f.

#### Etymology.

This new species is named after late Dr. Liang-Yih Chou (周樑鎰), who worked as Researcher at the TARI, conducted insect diversity projects during 1979–1984 and collected most of type series of this new species.

#### Distribution.

**Figure 7.**
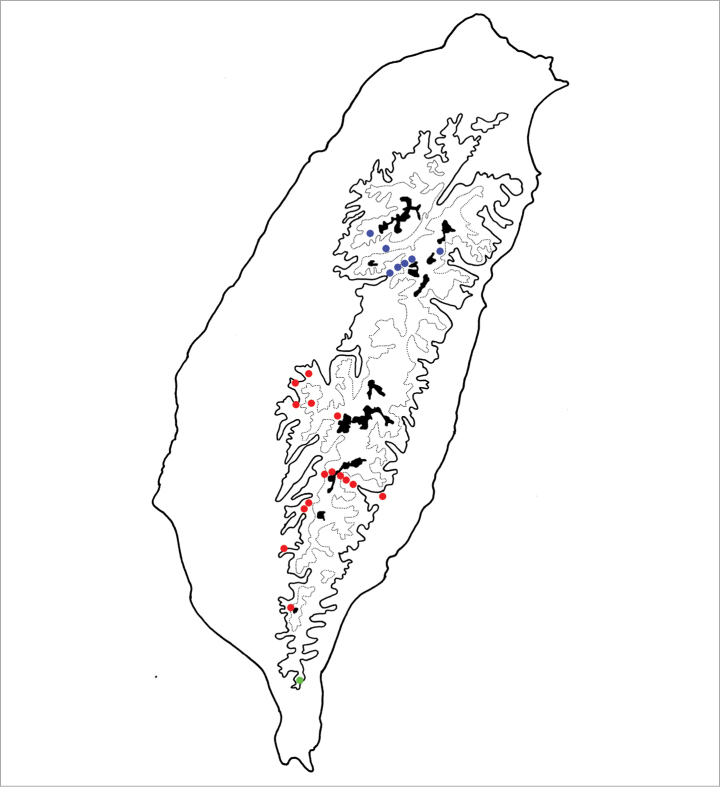
Distribution map of *Batophila* species, solid line: 1000 m a.s.l., broken line: 2000 m a.s.l., black areas: 3000 m a.s.l. Red dots *B. tsoui* sp. nov. Purple dots *B. choui* sp. nov. Green dot *B. chungi* sp. nov.

This species is widespread in mountainous areas of central Taiwan (Fig. 7).

### ﻿ Batophila chungi sp. nov.

Taxon classificationAnimaliaColeopteraChrysomelidae

0DBAFE99-18D4-5588-96A6-6B45D5A48BD6

https://zoobank.org/C28A03E4-F0BE-4E2E-B7D6-6E244EDEB403

Figs 7, 8

#### Type specimens examined (n = 20).

***Holotype*** ♂ (TARI): **Taiwan** • **Pingtung**: Tahanshan (大漢山), 26.II.2025, leg. Y.-T. Chung. ***Paratypes*** • 4♂♂, 6♀♀ (TARI), same data as holotype; • 1♂ (TARI), same but with “12.III.2025"; • 1♀ (TARI), same locality, 6.II.2008, leg. S.-F. Yu; • 1♂ (TARI), same locality, 14.VIII.2011, leg. Y.-T. Wang; • 1♀ (TARI), same locality, Malaise trap, 3.IV.–2.V.2020, leg. Y.-C. Chiu; • 3♂♂, 2♀♀ (TARI), same locality, 8.III.2025, leg. J.-C. Chen.

#### Diagnosis.

**Figure 8.**
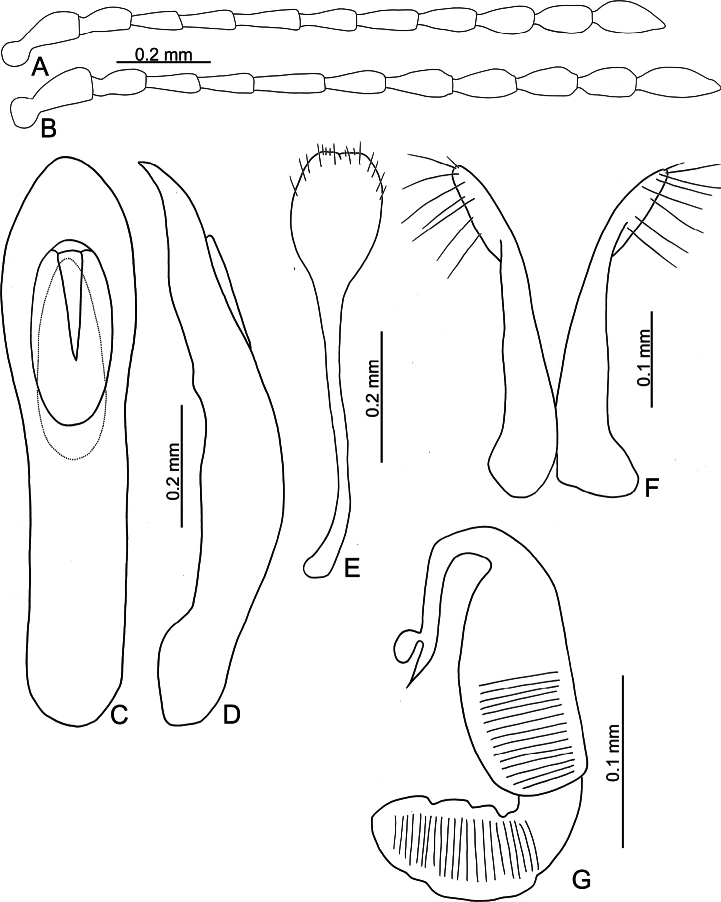
*Batophila chungi* sp. nov. A. Antenna, male; B. Antenna, female; C. Aedeagus, dorsal view; D. Aedeagus, lateral view; E. Abdominal ventrite VIII, female; F. Spermatheca; G. Gonocoxae.

Adults of *B. chungi* sp. nov., *B. tsoui* sp. nov., and *B. choui* sp. nov. are recognized by their strongly apically narrowed elytra, and divergent elytral apices, but *B. chungi* sp. nov. and *B. tsoui* sp. nov. differ in possessing flattened elytra in males and convex elytra in females (Fig. 21C, E) [convex elytra and elytral apex not visible in dorsal views in both sexes of *B. choui* sp. nov. (Fig. 5C, E)], and widened apex of aedeagus (Fig. 8C) [parallel-sided aedeagus in *B. choui* sp. nov. (Fig. 6C)]. Adults of *B. chungi* sp. nov. are not separable from those *B. tsoui* sp. nov. by external morphology but the aedeagus of *B. chungi* sp. nov. (Fig. 8C) is much wider than that of *B. tsoui* sp. nov. (Fig. 22C).

#### Description.

Male. Length 1.95–2.32 mm, width 0.79–0.91 mm. General color metallic dark bronze; legs yellowish but femora of hind legs darkened. Antenna (Fig. 8A) filiform and antennomeres VIII–X wide, ratio of length of antennomeres I–XI to length of antennomere I 1.0: 0.6: 0.6: 0.6: 0.8: 0.7: 0.7: 0.8: 0.7: 0.6: 0.9; ratio of length to width of antennomeres I–XI 2.3: 2.1: 2.7: 2.9: 3.3: 2.6: 2.2: 2.4: 2.0: 1.9: 2.3. Pronotum 1.10–1.12× wider than long; lateral margins slightly rounded, anterolateral angles separated from lateral margins by weak emarginations, slightly narrowed basally, distance between anterolateral angles 1.16–1.20× wider than basal margin. Elytra 1.57–1.60× longer than wide; lateral margins rounded, widest at basal 1/5, apically and strongly narrowed, apex truncate but divergent; dorsoventrally flattened, apex visible in dorsal view; disc with longitudinal lines of extremely coarse punctures and distinct longitudinal grooves along punctures, punctures and grooves apically abbreviated from apical 1/3. Tarsomeres I of front and middle legs slightly swollen. Aedeagus (Fig. 8C, D) elongate, 4.4× longer than wide; widest at apical 1/5, apically narrowed towards apex, apex widely rounded, basally widened near apical 2/5, then parallel-sided near base; dorsal opening starting from apical 1/7–9/20, tectum composed of three lobes, median lobe more basal relative to lateral lobes, apical margin truncate, mostly membranous; slightly curved in lateral view, apex moderately curved; ventral surface with membranous area as wide as dorsal opening, starting from apical 1/6–1/2.

Female. Length 2.11–2.45 mm, width 0.91–1.03 mm. Antennae similar to males, ratio of length of antennomeres I–XI to length of antennomere I (Fig. 8B) 1.0: 0.6: 0.6: 0.6: 0.8: 0.7: 0.8: 0.7: 0.7: 0.7: 1.0; ratio of length to width of antennomeres I–XI 2.6: 2.0: 2.7: 2.7: 3.6: 2.7: 2.3: 2.3: 2.1: 2.0: 2.9. Elytra 1.49–1.57× longer than wide; lateral margins rounded, widest at basal 1/5, apex truncate but divergent; dorsoventrally convex, apex not visible in dorsal view; disc with longitudinal lines of extremely coarse punctures and with distinct longitudinal grooves along punctures, punctures and grooves apically abbreviated from apical 1/3. Gonocoxae (Fig. 8F) slender, connected at basal 1/5; each gonocoxa with seven long setae and one tiny seta from apical 1/5 to apex, subapically slightly curved. Ventrite VIII (Fig. 8E) weakly sclerotized apically, with several short setae at apical area, and some tiny setae at apical margin, spiculum extremely elongate. Spermathecal receptaculum (Fig. 8G) strongly swollen, with transverse wrinkles at basal 1/2; pump wide and curved, with transverse wrinkles at apical 2/3; sclerotized spermathecal canal moderately long before base of spermathecal gland.

#### Food plants.

**Figure 9.**
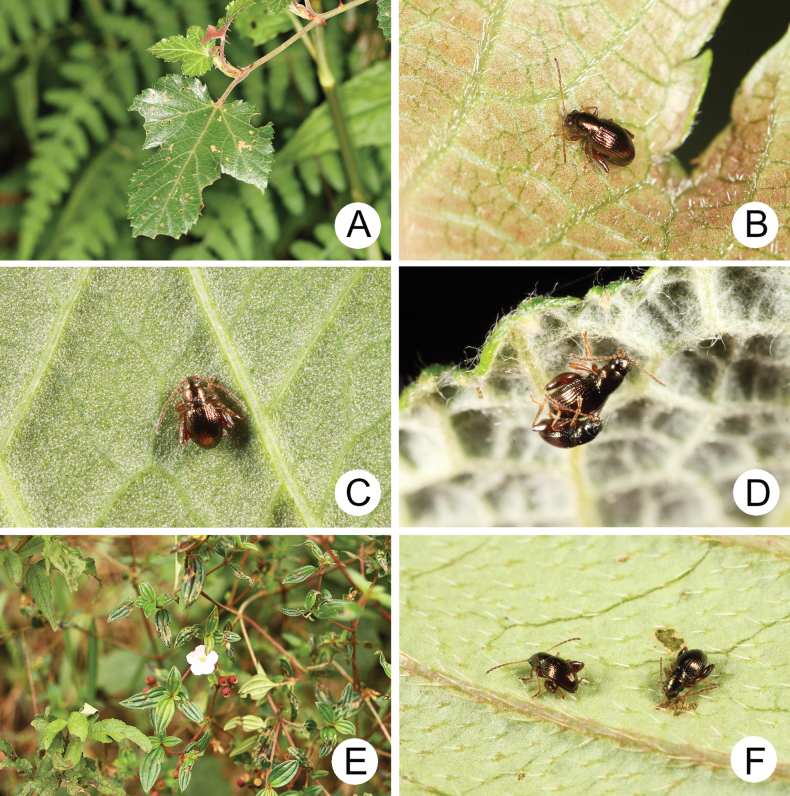
Field photographs of *Batophila* species A. Rubus taitoensis var. aculeatiflorus (Rosaceae); B. Adult of *B. chungi* sp. nov. on underside of leaf of R. taitoensis var. aculeatiflorus; C. Adult of *B. houjayi* sp. nov. resting on underside of leaf of *Persicaria chinense* (Polygonaceae); D. Adults of *B. tsoui* sp. nov. resting on underside of leaf of *R. wallichianus* (Rosaceae); E. *Otanthera scaberrima* (Melastomataceae); F. Adults of *B. tsoui* sp. nov. feeding on underside of leaf of *O. scaberrima*

Rosaceae: *Rubus formosensis* Kuntze; R. taitoensis var. aculeatiflorus (Hayata) H. Ohashi & C. F. Hsieh (Fig. 9A, B).

#### Etymology.

This new species is named for Yi-Ting Chung (鍾奕霆), the first member of TCRT to collect specimens.

#### Distribution.

Only known from the type locality in southern Taiwan (Fig. 7).

### ﻿ Batophila houjayi sp. nov.

Taxon classificationAnimaliaColeopteraChrysomelidae

D2119E9C-F283-50E2-8205-3CAEC537D261

https://zoobank.org/3A0988F9-87A4-46FA-A4A5-FF7719FA8A03

Figs 10, 11, 12, 13

Batophila acutangula : Kimoto 1989: 269 (part).
Batophila yangweii : Chûjô 1937: 54 (part).

#### Type specimens examined (n = 868).

***Holotype*** ♂ (TARI). **Taiwan** • **Nantou**: Tatachia (塔塔加), 21.IX.2009, leg. C.-F. Lee. ***Paratypes*** • 4♀♀ (TARI), same data as holotype; **Taiwan** • **Chiayi**: 1♂, 3♀♀ (KMNH), Alishan (阿里山), 21.V.1971, leg. K. Kamiya; • 4♂♂, 2♀♀ (KMNH), same but with “25.V.1971"; • 1♀ (KMNH), same but with “26.V.1971"; • 1♀ (KMNH), same locality, 22–25.VI.1974, leg. M. Owada; • 45♂♂, 30♀♀ (TARI), same locality, 5–9.VIII.1981, leg. L. Y. Chou & S. C. Lin; • 129♂♂, 56♀♀ (TARI), same locality, 17–20.VIII.1982, leg. K. C. Chou & C. C. Pan; • 1♀ (TARI), same locality, 25.IV.2009, leg. H.-J. Chen; • 3♂♂ (NMNS), same locality, 4.X.1988, leg. K. S. Huang; • 28♂♂, 10♀♀ (KMNH), same locality, 6.VIII.1990, leg. S. Kimoto, of which one male identified as *B. acutangula* by Kimoto in 1990; • 3♂♂, 1♀ (TARI), same locality, 12.V.2011, leg. C.-F. Lee; • 8♂♂, 4♀♀ (NHMUK), SE of Alishan, 23°28'20"N, 120°51'E, 2300 m, 6–8.XI.2008, leg. L. Dembický; • 2♂♂, 1♀ (TARI), Tzuchung (自忠), 21.IX.2009, leg. M.-H. Tsou; • 2♂♂ (TARI), same locality, 22.II.2016, leg. Y.-T. Wang; • **Hualien**: 16♂♂, 17♀♀ (TARI), Tayuling (大禹嶺), 9–16.VI.1980, leg. K. S. Lin & B. H. Chen; • 2♂♂, 2♀♀ (TARI), same locality and collectors, 10–16.VI.1980, Malaise trap; • 4♂♂, 8♀♀ (TARI), same locality, 12–15.IX.1980, leg. K. S. Lin & C. H. Wang; • 1♂, 1♀ (NHMUK), SW above Tayuling, 24°10'N, 121°17'30E, 2950 m, 31.X.2008, leg. L. Dembický; • **Ilan**: 1♀ (TARI), Sikikun (= Sunchitsun, 四季村), 11.VII.1933, leg. M. Chujo, identified as *B. yangweii* by Chûjô (1937); • 1♂, 2♀♀ (TARI), same locality (= Ssuchi), 7.VII.2009, leg. H.-J. Chen; • 1♂ (TARI), same but with “19.V.2010"; • 1♂ (TARI), Taiheizan (= Taipingshan, 太平山), 9.VII.1933, leg. M. Chujo, identified as *B. yangweii* by Chûjô (1937); • 5♂♂, 5♀♀ (TARI), same locality, 7.VII.1940, leg. R. Matuda; • **Kaohsiung**: 1♂, 1♀ (KMNH), Tienchi (天池), 2.V.1986, leg. K. Baba, identified as *B. acutangula* by Kimoto (1989); • **Nantou**: 1♀ (TARI), Huakang (華崗), 14.IX.2010, leg. C.-F. Lee; • 1♂, 1♀ (NMNS), Kahoershan (卡賀爾山), 7.V.1992. leg. W. T. Yang; • 1♂ (NMNS), Lienhuachih (蓮華池), 9.IV.–19.V.1998, leg. C. S. Lin & W. T. Yang; • 1♂ (KMNH), Lushan Wenchuan (廬山溫泉), 6.VI.1976, leg. H. Makihara; • 7♂♂, 16♀♀ (NMNS), Nanhuashan (南華山), 6.V.1992, leg. Yang & Huang; • 1♂ (NHMUK), (near Sungkang, 松崗) sheep farm, 24°03.121'N, 121°09.643'E, 1916 m, 7.VIII.2008, leg. M. V. L. Barclay & Mendel; • 1♂, 2♀♀ (TARI), Tatachia (塔塔加), 29.X.2009, leg. H. Lee; • 1♂ (TARI), same locality, 17.XI.2009, leg. C.-F. Lee; • 1♀ (TARI), same locality, 18.XI.2009, leg. H. Lee; • 3♂♂, 14♀♀ (TARI), Tsuifeng (翠峰), 21.VI.1979, leg. K. S. Lin & B. H. Chen; • 8♀♀ (TARI), same locality, 3.VI.1980, leg. L. Y. Chou & C. C. Chen; • 4♂♂, 35♀♀ (TARI), same locality, 25–27.VI.1981, leg. K. S. Lin & W. S. Tang; • 32♂♂, 90♀♀ (TARI), same locality, 1–3.VIII.1981, leg. T. Lin & W. S. Tang; • 1♂, 1♀ (TARI), same locality, 8.XI.1981, leg. S. C. Lin & W. S. Tang; • 2♂♂, 2♀♀ (TARI), same locality, 23.V.1982, leg. L. Y. Chou; • 33♂♂, 66♀♀ (TARI), same locality, 1–3.IX.1982, leg. L. Y. Chou & K. C. Chou; • 3♀♀ (TARI), same locality, 20.IV.1983, leg. K. C. Chou & S. P. Huang; • 1♀ (TARI), same locality, IV.1984, Malaise trap, leg. K. S. Lin & K. C. Chou; • 6♂♂, 17♀♀ (TARI), same locality, 9.V.1984, leg. K. C. Chou & C. C. Pan; • 6♂♂, 29♀♀ (TARI), same locality, 5.VIII.1984, leg. K. S. Lin; • 13♂♂, 26♀♀ (TARI), same locality, 12–14.IX.1984, leg. K. S. Lin & S. C. Lin; • 4♂♂, 1♀ (NMNS), Yuanfeng (鳶峰), 9.III.–9.IV.1998, leg. C. S. Lin & W. T. Yang, Malaise trap; • 5♂♂, 5♀♀ (NMNS), same but with “12.III.–9.IV.2002"; • 2♂♂, 2♀♀ (NMNS), same but with “9.IV.–7.V.2002"; • 2♀♀ (NMNS), same but with “11.VI.–9.VII.2002"; • 1♂, 2♀♀ (NMNS), same but with “17.IV.–7.V.2003"; • 1♀ (NMNS), same but with “11.VI.–8.VII.2003"; • 3♀♀ (NMNS), same but with “11.V.–13.VII.2004"; • 1♀ (NMNS), same but with “13.III.–10.IV.2007"; • 6♀♀ (NMNS), Yunhaipaohsienso (雲海保線所), 4.V.1992, leg. W. T. Yang; • 1♀ (KMNH), Yushan (玉山), 19.V.1981, leg. N. Ito, identified as *B. acutangula* by Kimoto in 1987.

#### Diagnosis.

Adults of *B. houjayi* sp. nov. are not separable from those of *B. wusheensis* sp. nov., *B. yuae* sp. nov., *B. jungchani* sp. nov., and *B. huangi* sp. nov. that are characterized by truncate elytral apices based on external morphology except for the aedeagus (see below). However, these species can be recognized by their allopatric distributions [*B. houjayi* sp. nov. inhabits high mountains in Chiayi, Ilan, Hualien, and Nantou counties, *B. wusheensis* sp. nov. in lowlands of Nantou County, *B. yuae* sp. nov. in lowlands of Taipei and New Taipei Cities, and Ilan County, *B. jungchani* sp. nov. in high mountains of Taichung and Miaoli counties, *B. huangi* sp. nov. in lowlands of Miaoli County and high mountains in Hsinchu and Taoyuan counties (Fig. 13)]. Aedeagal shapes are diagnostic [widely rounded apex of aedeagus in *B. houjayi* sp. nov. (Fig. 11C), apically tapering aedeagus from apical 1/5 in *B. wusheensis* sp. nov. (Fig. 23C), rounded apex of aedeagus with truncate process at middle of apical margin in *B. yuae* sp. nov. (Fig. 26C), subapically tapering apex of aedeagus in *B. jungchani* sp. nov. (Fig. 15C), and rounded apex of aedeagus with small, rounded process at middle of apical margin in *B. huangi* sp. nov. (Fig. 14C)].

#### Description.

**Figure 10.**
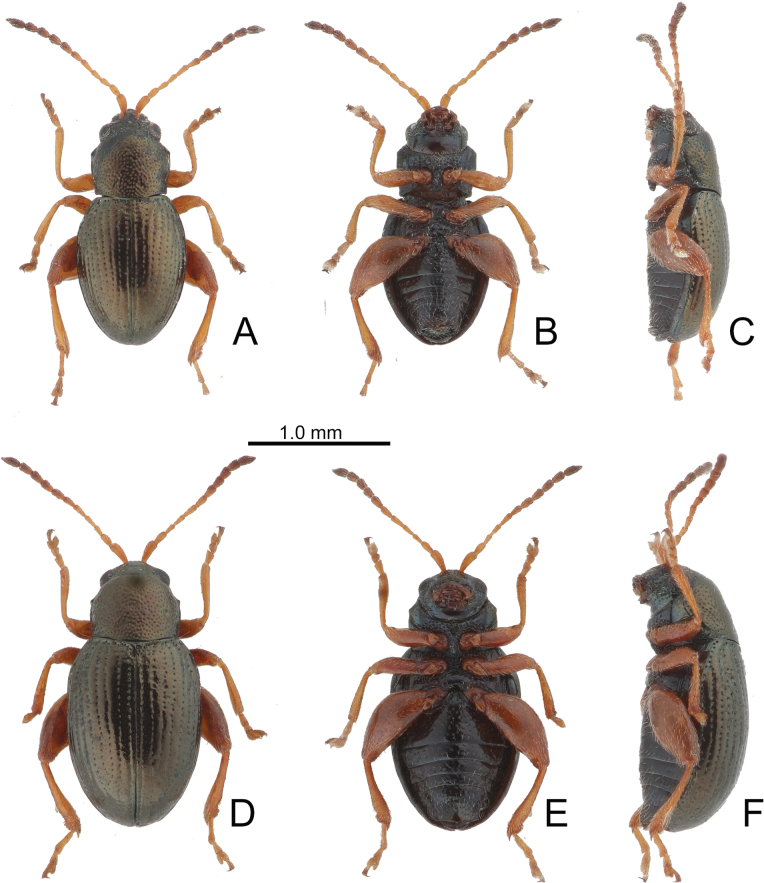
Habitus of *Batophila houjayi* sp. nov., from Tsuifeng (翠峰) A. Male, paratype, dorsal view; B. Ditto, ventral view; C. Ditto, lateral view; D. Female, paratype, dorsal view; E. Ditto, ventral view; F. Ditto, lateral view.

**Figure 11.**
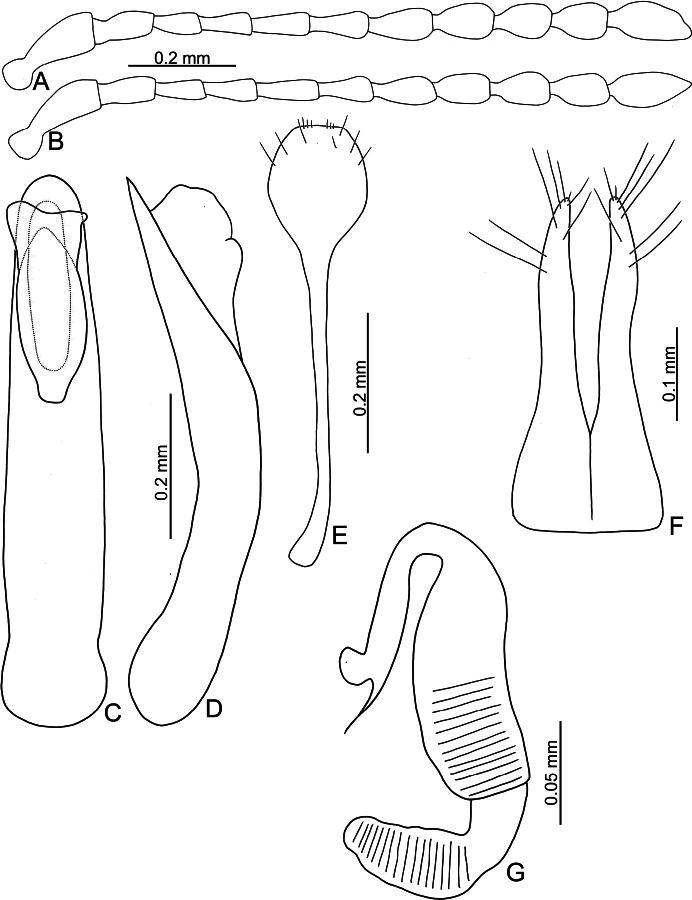
*Batophila houjayi* sp. nov. A. Antenna, male; B. Antenna, female; C. Aedeagus, dorsal view; D. Aedeagus, lateral view; E. Abdominal ventrite VIII, female; F. Spermatheca; G. Gonocoxae.

Male. Length 1.63–1.92 mm, width 0.82–0.94 mm. General color metallic dark bronze (Fig. 10A–C); antennae yellowish brown but six apical antennomeres darker; legs yellowish but femora of hind legs darkened. Antenna (Fig. 11A) filiform and antennomeres VIII–X wide, ratio of length of antennomeres I–XI to length of antennomere I 1.0: 0.6: 0.5: 0.5: 0.6: 0.6: 0.6: 0.6: 0.6: 0.6: 0.8; ratio of length to width of antennomeres I–XI 2.6: 2.1: 2.2: 2.3: 2.6: 2.2: 2.0: 1.8: 1.7: 1.6: 2.3. Pronotum 1.22–1.30× wider than long; lateral margins slightly rounded, anterolateral angles separated from lateral margins by weak emarginations, slightly narrowed basally, distance between anterolateral angles 1.10–1.17× wider than basal margin. Elytra 1.28–1.34× longer than wide; lateral margins rounded, widest at basal 1/5, apex truncate; dorsoventrally flattened, apex visible in dorsal view; disc with longitudinal lines of coarse punctures, and distinct longitudinal grooves along punctures apically abbreviated, ridges present between longitudinal grooves and apically abbreviated from basal 1/3. Tarsomeres I of front and middle legs slightly swollen. Aedeagus (Fig. 11C, D) elongate, 5.7× longer than wide; lateral margins subapically parallel, then slightly widened basally, widest at basal 1/3, apex widely rounded; dorsal opening starting from apical 1/10–2/5, tectum membranous; moderately curved in lateral view; ventral surface with membranous area narrower than dorsal opening, starting from apical 1/20–1/3.

Females (Fig. 10D–F). Length 1.79–2.21 mm, width 0.96–1.10 mm. Antennae similar to males, ratio of length of antennomeres I–XI to length of antennomere I (Fig. 11B) 1.0: 0.6: 0.5: 0.5: 0.6: 0.5: 0.6: 0.6: 0.6: 0.6: 0.8; ratio of length to width of antennomeres I–XI 2.9: 2.2: 2.5: 2.2: 3.0: 2.3: 2.2: 1.8: 1.8: 1.7: 2.4. Elytra 1.26–1.38× longer than wide; lateral margins rounded, widest at basal 1/5, apex truncate; dorsoventrally convex, elytral apex not visible in dorsal view; disc with longitudinal lines of fine punctures, and distinct longitudinal grooves along punctures apically abbreviated from apical 1/3, ridges present between 2^nd^ and 3^rd^, 4^th^ and 5^th^ longitudinal grooves and apically abbreviated from basal 1/3. Gonocoxae (Fig. 11F) slender, connected from basal 1/5 to base; each gonocoxa with seven long setae and one tiny seta from apical 1/5 to apex, subapically slightly curved. Ventrite VIII (Fig. 11E) weakly sclerotized apically, with several short setae at sides of apex, and some tiny setae at sides of apical margin, spiculum extremely elongate. Spermathecal receptaculum (Fig. 11G) strongly swollen, with transverse wrinkles at basal 1/2; pump wide and curved, with transverse wrinkles at apical 2/3; sclerotized spermathecal canal moderately long before base of spermathecal gland.

#### Variation.

**Figure 12.**
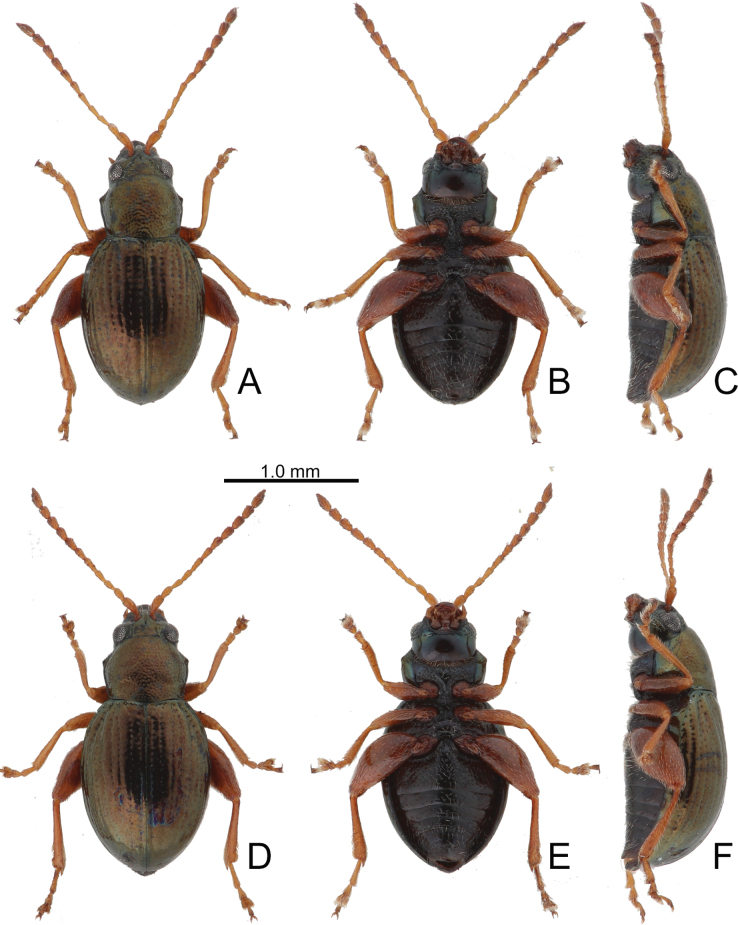
Habitus of *Batophila houjayi* sp. nov., from Alishan (阿里山) A. Male, paratype, dorsal view; B. Ditto, ventral view; C. Ditto, lateral view; D. Female, paratype, dorsal view; E. Ditto, ventral view; F. Ditto, lateral view.

Longitudinal ridges on elytra present in most individuals from Alishan (Fig. 12) but absent in specimens from other areas.

**Figure 13.**
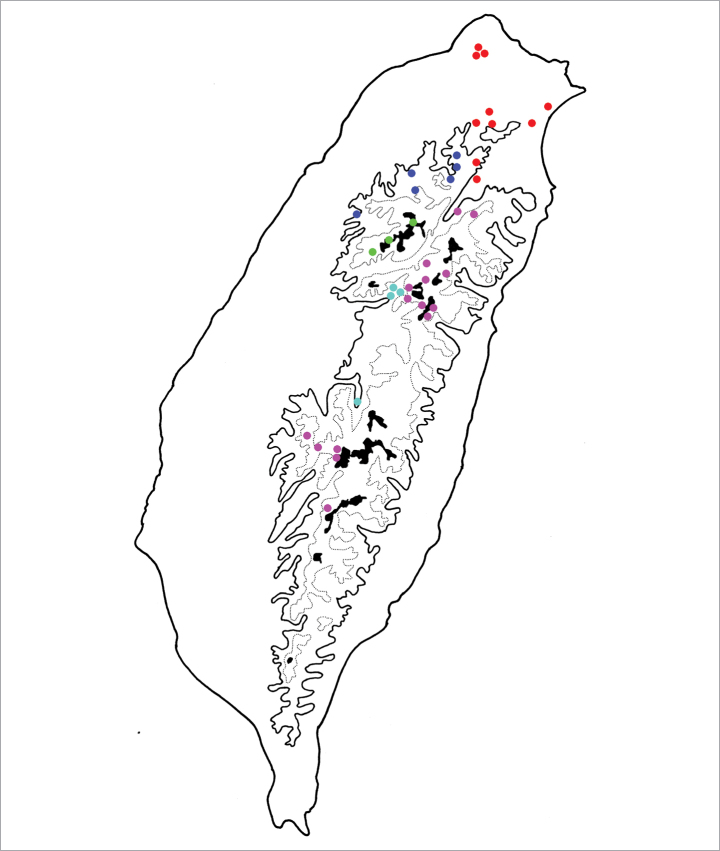
Distribution map of *Batophila* species, solid line: 1000 m a.s.l., broken line: 2000 m a.s.l., black areas: 3000 m a.s.l. Red dots *B. yuae* sp. nov. Blue dots *B. wusheensis* sp. nov. Green dot *B. jungchani* sp. nov. Pink dots *B. houjayi* sp. nov. Purple dots *B. huangi* sp. nov.

**Figure 14.**
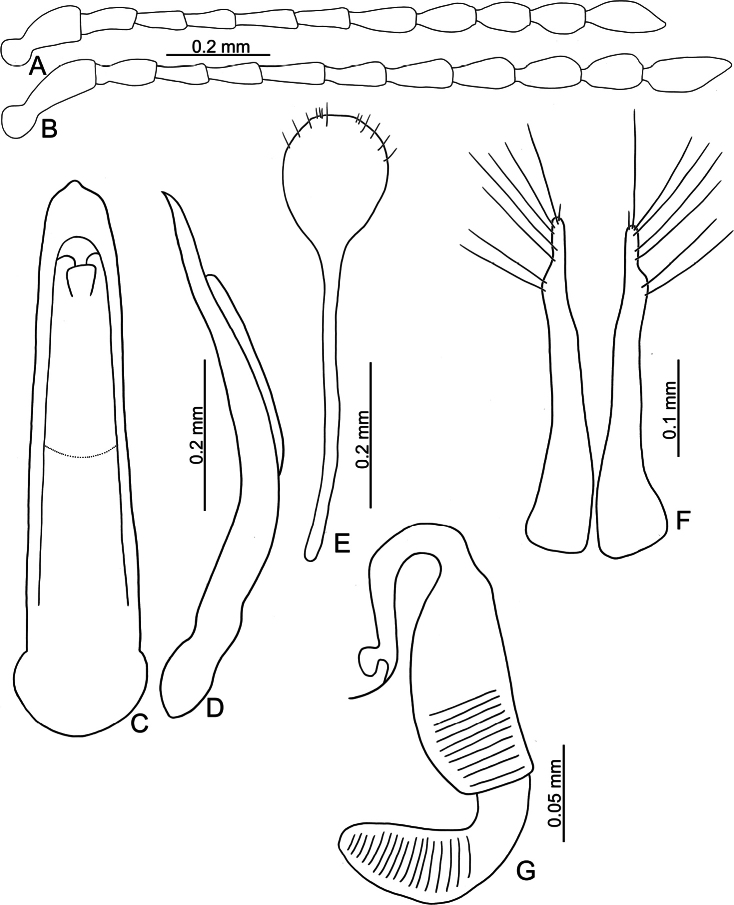
*Batophila huangi* sp. nov. A. Antenna, male; B. Antenna, female; C. Aedeagus, dorsal view; D. Aedeagus, lateral view; E. Abdominal ventrite VIII, female; F. Spermatheca; G. Gonocoxae.

#### Food plants.

Rosaceae: *Rubus lambertianus* Ser. and *R. croceacanthus* H. Lév.; Polygonaceae: *Persicaria thunbergia* Sieb. et Zucc. and *P. chinense* L. (Fig. 9C).

#### Etymology.

This new species is named for Hou-Jay Chen (陳厚潔), the first member of TCRT to collect specimens.

#### Distribution.

This species is widespread in mountainous areas of south and central Taiwan (Fig. 13).

### ﻿ Batophila huangi sp. nov.

Taxon classificationAnimaliaColeopteraChrysomelidae

C4F8F5ED-3220-52F5-80B4-713F6D5456B7

https://zoobank.org/3D4EAF0C-A47A-43F0-94D2-9AE69C825FBD

Fig. 14

Batophila yangweii : Chûjô 1937: 54 (part).

#### Type specimens examined (n = 22).

***Holotype*** ♂ (NMNS). **Taiwan** • **Miaoli**: Taian (泰安), 20.XII.1989, leg. K. W. Huang. ***Paratypes*** • 8♂♂, 3♀♀ (NMNS), same data as holotype; **Hsinchu**: • 2♂♂ (TARI), Talulintao (大鹿林道), 17.II.2008, leg. M.-H. Tsou; 1♂ (TARI), Wufeng (五峰), 14–16.VII.1982, leg. K. C. Chou & C. C. Pan; • 1♂ (NMNS), same locality, 21.XII.1989, leg. K. W. Huang; • **Taoyuan**: 1♂ (TARI), Kayahara (= Hsuanyuan, 萱源), 23.VII.1929, leg. Y. Miwa, identified as *B. yangweii* by Chûjô (1937); • 1♂, 1♀ (TARI), Lalashan (拉拉山), 8.III.2009, leg. H. Lee; • 1♂ (TARI), same but with “leg. H.-J. Chen”; • 1♀ (TARI), same but with “leg. C.-F. Lee”; • 1♀ (NMNS), Upper Plain (sic!) (= Balung, 上巴陵), 24°41'12.1"N, 121°23'39.3"E, 600 m, 11.IV.1998, leg. Miller, Stange, Wang.

#### Diagnosis.

**Figure 15.**
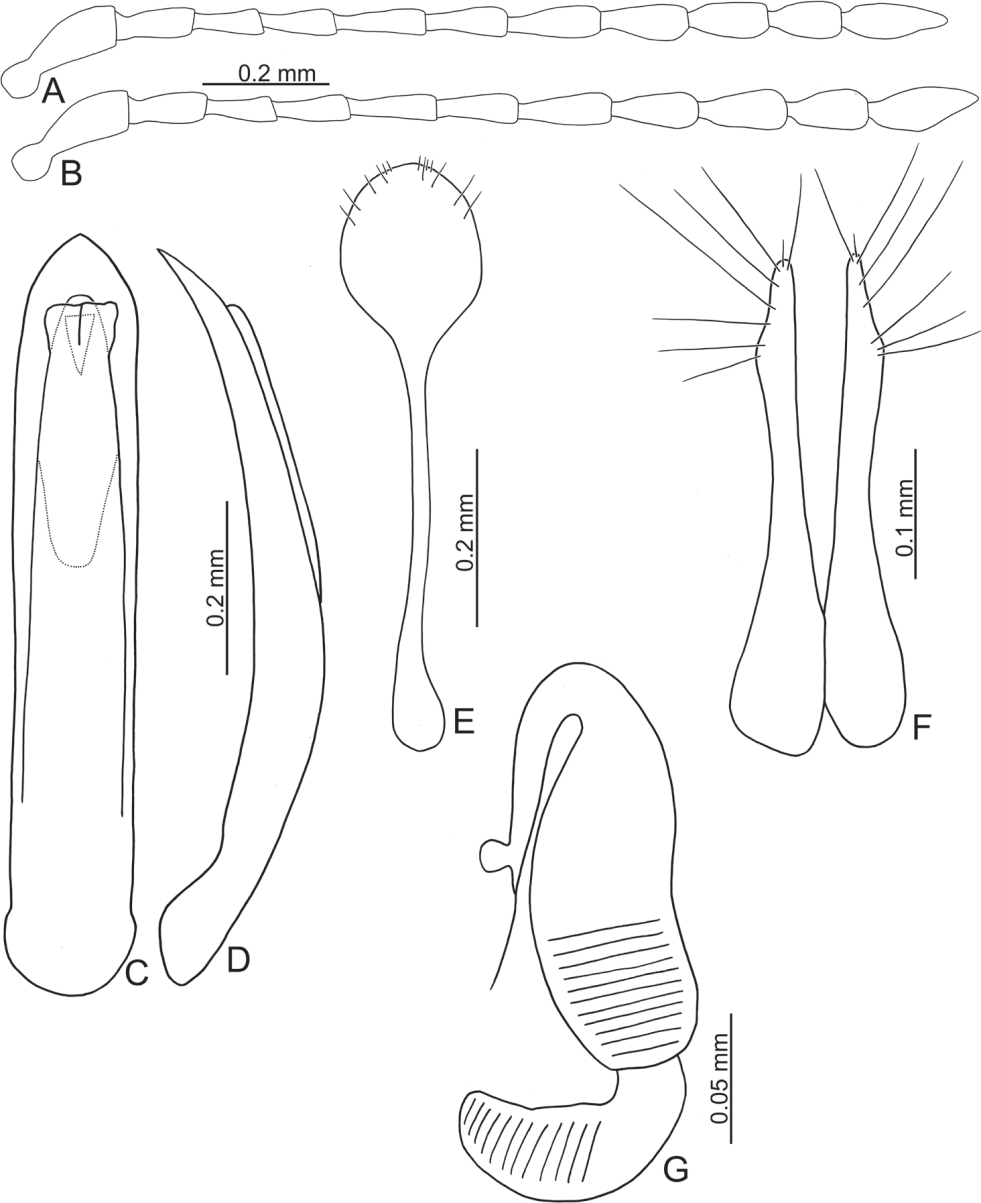
*Batophila jungchani* sp. nov. A. Antenna, male; B. Antenna, female; C. Aedeagus, dorsal view; D. Aedeagus, lateral view; E. Abdominal ventrite VIII, female; F. Spermatheca; G. Gonocoxae.

Adults of *B. huangi* sp. nov. are not separable from those of *B. houjayi* sp. nov., *B. wusheensis* sp. nov., *B. yuae* sp. nov., and *B. jungchani* sp. nov. that are characterized by truncate elytral apices based on external morphology (Figs 10, 12) except for the aedeagus (see below). However, these species can be recognized by their allopatric distributions [*B. huangi* sp. nov. inhabits lowlands in Miaoli County and high mountains in Hsinchu and Taoyuan counties, *B. jungchani* sp. nov. in high mountains in Taichung and Miaoli counties, *B. yuae* sp. nov. in lowlands of Taipei and New Taipei Cities, and Ilan County, *B. wusheensis* sp. nov. in lowlands of Nantou County, *B. houjayi* sp. nov. at high mountains in Chiayi, Ilan, Hualien, and Nantou counties (Fig. 13)]. Aedeagal shapes are diagnostic [rounded apex of aedeagus with small, rounded process at middle of apical margin in *B. huangi* sp. nov. (Fig. 14C), apically tapering apex of aedeagus from apical 1/10 in *B. jungchani* sp. nov. (Fig. 15C), rounded apex of aedeagus with truncate process at middle of apical margin in *B. yuae* sp. nov. (Fig. 26C), apically tapering aedeagus from apical 1/5 in *B. wusheensis* sp. nov. (Fig. 23C), and widely rounded apex of aedeagus in *B. houjayi* sp. nov. (Fig. 11C)].

#### Description.

Male. Length 1.52–1.75 mm, width 0.77–0.86 mm. General color metallic dark bronze; antennae yellowish brown but six apical antennomeres darker; legs yellowish but femora of hind legs darkened. Antenna (Fig. 14A) filiform and antennomeres VIII–X wide, ratio of length of antennomeres I–XI to length of antennomere I 1.0: 0.6: 0.6: 0.6: 0.7: 0.6: 0.8: 0.7: 0.6: 0.6: 0.9; ratio of length to width of antennomeres I–XI 2.4: 2.5: 2.5: 2.5: 3.2: 2.5: 2.7: 2.1: 1.8: 1.8: 2.7. Pronotum 1.18–1.25× wider than long; lateral margins slightly rounded, anterolateral angles separated from lateral margins by weak emarginations, slightly narrowed basally, distance between anterolateral angles 1.17–1.18× wider than basal margin. Elytra 1.35–1.37× longer than wide; lateral margins rounded, widest at basal 1/5, apex truncate; dorsoventrally flattened, apex visible in dorsal view; disc with longitudinal lines of coarse punctures, and indistinct longitudinal grooves along punctures, lacking ridges present between longitudinal grooves. Tarsomeres I of front and middle legs slightly swollen. Aedeagus (Fig. 14C, D) elongate, 5.3× longer than wide; lateral margins basally and slightly widened, widest near base, apex widely rounded and with small rounded process at middle of apical margin; dorsal opening starting from apical 1/10, basally weakly sclerotized; tectum composed of three lobes, median lobe more ventral relative to lateral lobes and apical margin truncate, mostly membranous; moderately curved subapically and medially in lateral view; ventral surface with membranous area same width and height as dorsal opening, starting from apical 1/10–1/2.

Female. Length 1.97–2.19 mm, width 1.00–1.06 mm. Antennae similar to males, ratio of length of antennomeres I–XI to length of antennomere I (Fig. 14B) 1.0: 0.6: 0.5: 0.5: 0.6: 0.6: 0.6: 0.6: 0.6: 0.6: 0.8; ratio of length to width of antennomeres I–XI 3.2: 2.3: 2.6: 2.6: 3.0: 2.6: 2.3: 2.1: 2.1: 1.8: 2.6. Elytra 1.36–1.39× longer than wide; lateral margins rounded, widest at basal 1/5, apex truncate; dorsoventrally convex, apex not visible in dorsal view; disc with longitudinal lines of coarse punctures, and indistinct longitudinal grooves along punctures, lacking ridges present between longitudinal grooves. Gonocoxae (Fig. 14F) slender, connected from basal 1/5 to base; each gonocoxa with seven long setae and one tiny seta from apical 1/5 to apex, subapically slightly curved. Ventrite VIII (Fig. 14E) weakly sclerotized apically, with several short setae at sides of apex, and some tiny setae at sides of apical margin, spiculum extremely elongate. Spermathecal receptaculum (Fig. 14G) strongly swollen, with transverse wrinkles at basal 1/2; pump wide and curved, with transverse wrinkles at apical 2/3; sclerotized spermathecal canal moderately long before base of spermathecal gland.

#### Food plants.

Rosaceae: *Rubus* sp.

#### Etymology.

This new species is named for Dr. Kun-Wei Huang (黃坤煒), who was a former research scientist at the NMNS and collected most of the type specimens.

#### Distribution.

This species is widespread in mountainous areas of northwestern Taiwan (Fig. 13).

### ﻿ Batophila jungchani sp. nov.

Taxon classificationAnimaliaColeopteraChrysomelidae

3BC830BB-A2E5-5658-AA33-1797188417E2

https://zoobank.org/0B81AB2D-59CC-4E7E-9152-21A4EA66F560

Fig. 15

#### Type specimens examined (n = 36).

***Holotype*** ♂ (NMNS). **Taiwan** • **Taichung**: Anmashan (鞍馬山), 3.V.1992, leg. C. Y. Li. ***Paratypes*** • 3♂♂, 12♀♀ (NMNS), same data as holotype; • 1♂, 1♀ (NMNS), same locality, 3.V.1990, leg. C. C. Chiang; • 5♀♀ (TARI), same locality, 21.IV.2010, leg. C.-F. Lee; • **Miaoli**: 1♂ (TARI), Hsiaopangchih (小胖池), 28.VIII.2021, leg. Y.-F. Hsu; • 4♂♂, 4♀♀ (TARI), same but with “16.XI.2021–21.IV.2022"; • **Taichung**: 2♂♂ (TARI), Hsuehshan (雪山), 24°23'15"N, 121°11'55"E, 29.IV.–28.VI.2012, leg. L.-P. Hsu; • 2♀♀ (TARI), Tahsuehshan (大雪山), 18.IV.2011, leg. J.-C. Chen.

#### Diagnosis.

Adults of *B. jungchani* sp. nov. are not separable from those of *B. houjayi* sp. nov., *B. wusheensis* sp. nov., *B. yuae* sp. nov., and *B. huangi* sp. nov. that are characterized by truncate elytral apices based on external morphology (Figs 10, 12) except for the aedeagus (see below). However, these species can be recognized by their allopatric distributions [*B. jungchani* sp. nov. inhabits high mountains in Taichung and Miaoli counties, *B. yuae* sp. nov. in lowlands of Taipei and New Taipei Cities, and Ilan County, *B. wusheensis* sp. nov. in lowlands of Nantou County, *B. houjayi* sp. nov. in high mountains in Chiayi, Ilan, Hualien, and Nantou counties, *B. huangi* sp. nov. in lowlands of Miaoli County and high mountains of Hsinchu and Taoyuan counties (Fig. 13)]. Aedeagal shapes are diagnostic [apically tapering aedeagus from apical 1/10 in *B. jungchani* sp. nov. (Fig. 15C), rounded apex of aedeagus with truncate process at middle of apical margin in *B. yuae* sp. nov. (Fig. 26C), apically tapering aedeagus from apical 1/5 in *B. wusheensis* sp. nov. (Fig. 23C), widely rounded apex of aedeagus in *B. houjayi* sp. nov. (Fig. 11C), and rounded apex of aedeagus with small, rounded process at middle of apical margin in *B. huangi* sp. nov. (Fig. 14C)].

#### Description.

Male. Length 1.93–2.01 mm, width 0.94–0.97 mm. General color metallic dark bronze; antennae and legs reddish brown. Antenna (Fig. 15A) filiform and antennomeres VIII–X wide, ratio of length of antennomeres I–XI to length of antennomere I 1.0: 0.6: 0.6: 0.6: 0.7: 0.7: 0.8: 0.7: 0.7: 0.6: 0.9; ratio of length to width of antennomeres I–XI 2.7: 2.3: 2.6: 2.4: 3.0: 2.7: 2.4: 2.3: 2.2: 2.1: 3.0. Pronotum 1.22–1.25× wider than long; lateral margins slightly rounded, anterolateral angles separated from lateral margins by weak emarginations, slightly narrowed basally, distance between anterolateral angles 1.14–1.15× wider than basal margin. Elytra 1.31–1.35× longer than wide; lateral margins rounded, widest at basal 1/5, apex truncate; dorsoventrally flattened, apex visible in dorsal view; disc with longitudinal lines of coarse punctures, and distinct longitudinal grooves along punctures, punctures and grooves apically abbreviated, lacking ridges present between longitudinal grooves. Tarsomeres I of front and middle legs slightly swollen. Aedeagus (Fig. 15C, D) elongate, 6.4× longer than wide; parallel-sided, apically tapering from apical 1/10; moderately curved in lateral view; dorsal opening starting from apical 1/15, basally weakly sclerotized; tectum composed of three lobes, median lobe more ventral relative to lateral lobes and apical margin truncate, mostly membranous; moderately curved subapically and medially in lateral view; ventral surface with membranous area same width and height as dorsal opening, starting from apical 1/15 to 1/3.

Female. Length 2.20–2.34 mm, width 1.06–1.17 mm. Antennae similar to males, ratio of length of antennomeres I–XI to length of antennomere I (Fig. 15B) 1.0: 0.6: 0.5: 0.6: 0.7: 0.6: 0.7: 0.7: 0.7: 0.6: 0.9; ratio of length to width of antennomeres I–XI 2.9: 2.5: 2.6: 3.0: 3.6: 2.8: 2.9: 2.5: 2.2: 2.0: 2.6. Elytra 1.32–1.38× longer than wide; lateral margins rounded, widest at basal 1/5, apex truncate; dorsoventrally convex, apex not visible in dorsal view; disc with longitudinal lines of coarse punctures, and indistinct longitudinal grooves along punctures, lacking ridges present between longitudinal grooves. Gonocoxae (Fig. 15F) slender, connected from basal 1/5 to base; each gonocoxa with seven long and one tiny setae from apical 1/5 to apex, subapically slightly curved. Ventrite VIII (Fig. 15E) weakly sclerotized apically, with several short setae at sides of apex, and some tiny setae at sides of apical margin, spiculum extremely elongate. Spermathecal receptaculum (Fig. 15G) strongly swollen, with transverse wrinkles at basal 1/2; pump wide and curved, with transverse wrinkles at apical 2/3; sclerotized spermathecal canal moderately long before base of spermathecal gland.

#### Variation.

**Figure 16.**
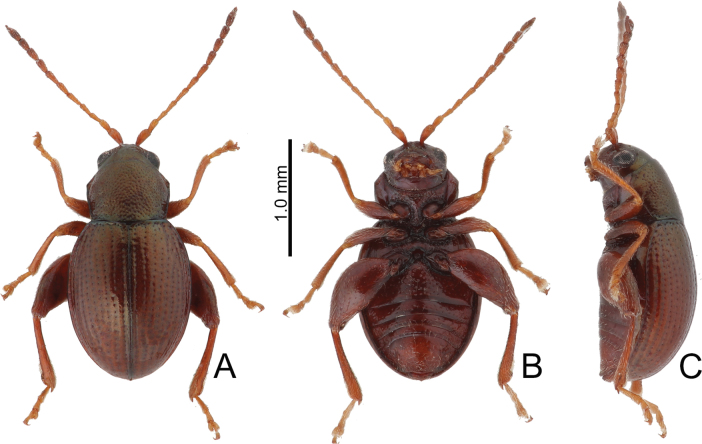
Habitus of *Batophila jungchani* sp. nov., female, paratype, from Anmashan (鞍馬山) A. Dorsal view; B. Ventral view; C. Lateral view.

Some individuals have reddish-brown elytra, especially from Anmashan (鞍馬山) (Fig. 16).

#### Food plants.

Unknown.

#### Etymology.

This new species is named for Jung-Chan Chen (陳榮章), the first person to collect specimens.

#### Distribution.

This species is widespread in alpine areas of central Taiwan (Fig. 13).

### ﻿ Batophila meihuai sp. nov.

Taxon classificationAnimaliaColeopteraChrysomelidae

AF39B7D6-497A-5AA2-B631-3B1A91E16B74

https://zoobank.org/6CB4AAAB-B575-4854-ACBF-FCA0BD610C15

Fig. 17, 18

#### Type specimens examined (n = 14).

***Holotype*** ♂ (TARI). **Taiwan** • **Ilan**: Ssuyuan (思源), 25.IV.2009, leg. C.-F. Lee. ***Paratypes*** • 2♂♂, 1♀ (TARI), same data as holotype; • 4♂♂, 4♀♀ (TARI), same locality, 28.IV.2009, leg. M.-H. Tsou; • 1♂♂ (TARI), same locality, 31.VII.2009, leg. H.-J. Chen; • **Taichung**: 1♂♂ (NMNS), same locality (= Ssuyuanyakou, 思源啞口), 25.VI.2007, leg. P. H. Chan & W. L. Lien. The type locality “Ssuyuan” is located at the border between Ilan County and Taichung County.

#### Diagnosis.

**Figure 17.**
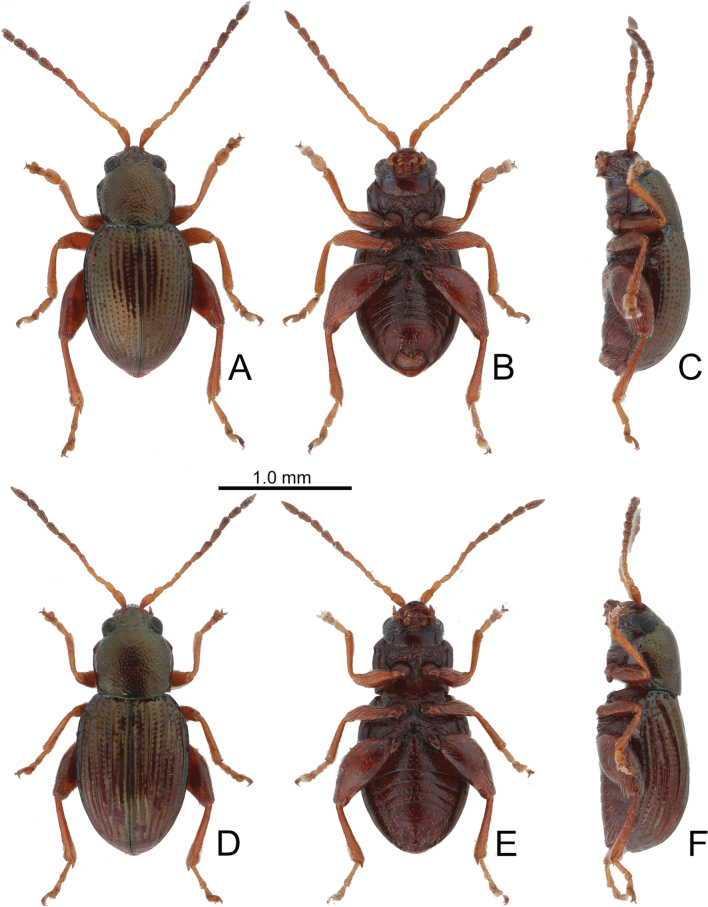
Habitus of *Batophila meihuai* sp. nov. A. Male, paratype, dorsal view; B. Ditto, ventral view; C. Ditto, lateral view; D. Female, paratype, dorsal view; E. Ditto, ventral view; F. Ditto, lateral view.

**Figure 18.**
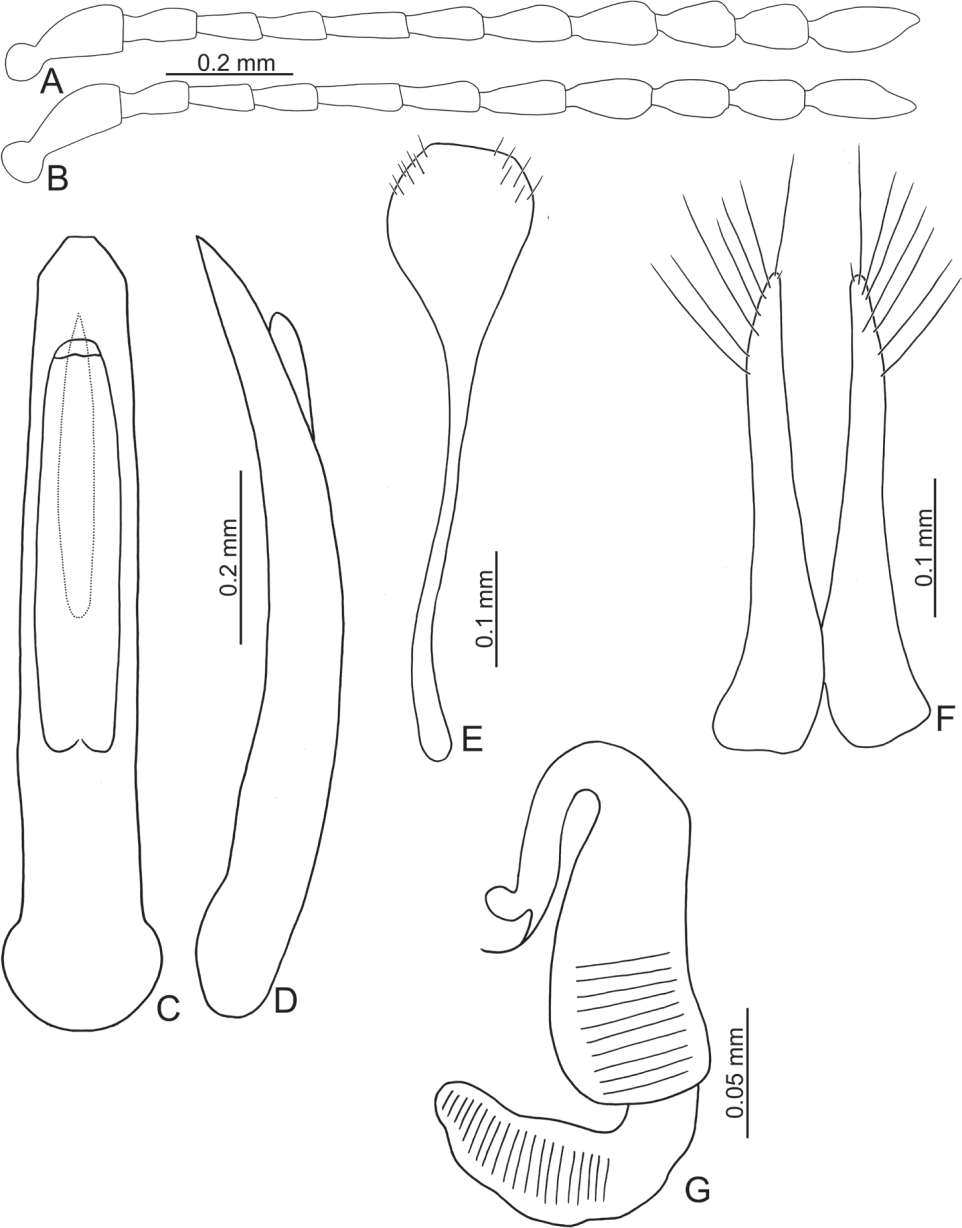
*Batophila meihuai* sp. nov. A. Antenna, male; B. Antenna, female; C. Aedeagus, dorsal view; D. Aedeagus, lateral view; E. Abdominal ventrite VIII, female; F. Spermatheca; G. Gonocoxae.

**Figure 19.**
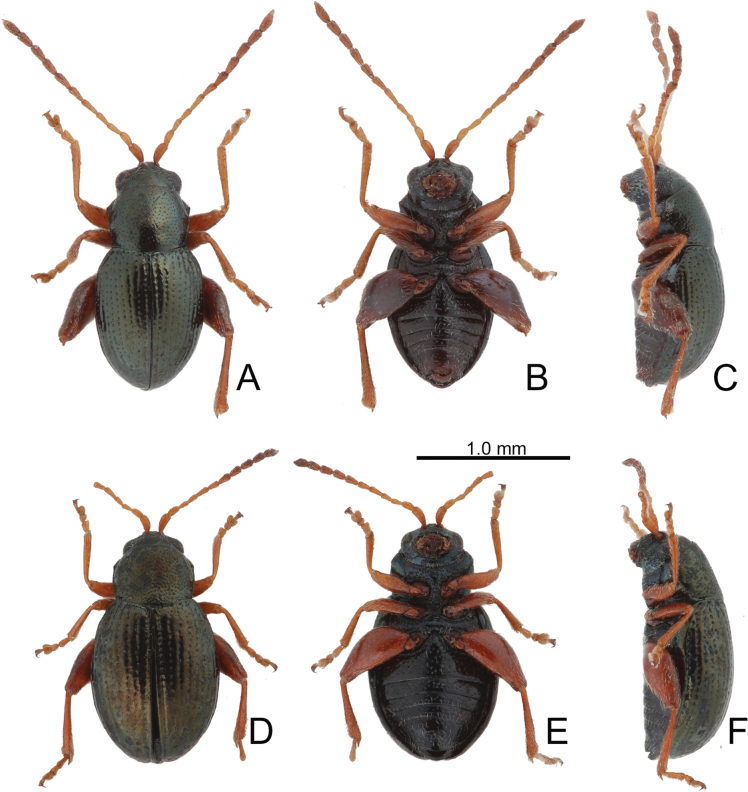
Habitus of *Batophila taiwanica* Döberl A. Male, dorsal view; B. Ditto, ventral view; C. Ditto, lateral view; D. Female, dorsal view; E. Ditto, ventral view; F. Ditto, lateral view.

Adults of *B. meihuai* sp. nov. are similar to those of *B. taiwanica* Döberl and *B. yehi* sp. nov. in possessing convergent elytral apices. However, adults of *B. yehi* sp. nov. are recognized by their stout antennae, length of antennomeres VI–X 0.5× length of antennomere I (Fig. 15A, B) [> 0.5× in *B. taiwanica* (Fig. 22A, B) and *B. meihuai* sp. nov. (Fig. 18A, B)]. Adults of *B. meihuai* sp. nov. are also characterized by their distinct and sexually dimorphic longitudinal ridges on the elytra (Fig. 17) [indistinct or reduced longitudinal ridges on the elytra in *B. taiwanica* (Fig. 19) and *B. yehi* sp. nov. (Fig. 24)]. The aedeagus of these species are diagnostic: truncate apex in *B. meihuai* sp. nov. (Fig. 18C), rounded apex with narrowly rounded process at middle of apical margin in *B. taiwanica* (Fig. 22C), and widely rounded apex in *B. yehi* sp. nov. (Fig. 25C).

#### Description.

Male. Length 1.64–2.08 mm, width 0.76–0.88 mm. General color metallic dark bronze (Fig. 17A–C); antennae yellowish brown but six apical antennomeres darker; legs yellowish but femora of hind legs apically darkened. Antenna (Fig. 18A) filiform and antennomeres VIII–X wide, ratio of length of antennomeres I–XI to length of antennomere I 1.0: 0.6: 0.5: 0.5: 0.7: 0.6: 0.7: 0.7: 0.6: 0.6: 0.9; ratio of length to width of antennomeres I–XI 2.5: 2.3: 2.3: 2.2: 3.2: 2.3: 2.4: 2.0: 1.7: 1.6: 2.5. Pronotum 1.19–1.32× wider than long; lateral margins straight, anterolateral angles not separated from lateral margins, slightly narrowed basally, distance between anterolateral angles widest, 1.12–1.14× wider than basal margin. Elytra 1.40–1.56× longer than wide; lateral margins parallel between basal 1/5 to apical 1/3, apex convergent; dorsoventrally flattened, apex visible in dorsal view; disc with longitudinal lines of coarse punctures, and longitudinal grooves along punctures, distinct ridges present between longitudinal grooves, apices of ridges rounded, apically abbreviated. Tarsomeres I of front and middle legs strongly swollen. Aedeagus (Fig. 18C, D) elongate, 6.9× longer than wide; parallel-sided, strongly tapering near apex, apical margin truncate; dorsal opening from apical 1/7 to basal 1/3, tectum membranous; slightly curved in lateral view; ventral surface with membranous area narrower than dorsal opening, starting from apical 1/8 to 1/2.

Female (Fig. 17D–F). Length 1.96–2.16 mm, width 0.90–1.03 mm. Antennae similar to males, but antennomeres VIII–X narrower than those of females, ratio of length of antennomeres I–XI to length of antennomere I (Fig. 18B) 1.0: 0.5: 0.5: 0.5: 0.6: 0.6: 0.6: 0.6: 0.6: 0.6: 0.8; ratio of length to width of antennomeres I–XI 2.8: 2.2: 2.4: 2.4: 3.0: 2.6: 2.3: 2.2: 2.1: 1.9: 2.8. Elytra 1.42–1.43× longer than wide; lateral margins parallel between basal 1/5 to apical 1/3, apex convergent; dorsoventrally convex, apex not visible in dorsal view; disc with longitudinal lines of coarse punctures, and longitudinal grooves along punctures, distinct ridges present between longitudinal grooves, apices of ridges acute, apically abbreviated. Gonocoxae (Fig. 18F) slender, connected from basal 1/5 to base; each gonocoxa with seven long setae and one tiny seta from apical 1/6 to apex, subapically slightly curved. Ventrite VIII (Fig. 18E) weakly sclerotized apically, with several short setae at sides of apex, spiculum extremely elongate. Spermathecal receptaculum (Fig. 18G) strongly swollen, with transverse wrinkles at basal 1/2; pump wide and curved, with transverse wrinkles at apical 2/3; sclerotized spermathecal canal moderately long before base of spermathecal gland.

#### Food plants.

Rosaceae: *Rubus* sp.

#### Etymology.

This new species is named for Mei-Hua Tsou (曹美華), the first member of TCRT to collect specimens.

#### Distribution.

Only known from the type locality in northeast Taiwan (Fig. 4).

### ﻿ Batophila taiwanica

Taxon classificationAnimaliaColeopteraChrysomelidae

Döberl, 2010

205D69B6-4FD0-593F-A469-746B9F2787E2

Figs 19, 20

Batophila acutangula : Kimoto 1971: 73 (part); Kimoto 1989: 269 (part).
Batophila yangweii : Chûjô 1937: 54 (part).

#### Type specimen examined.

***Holotype*** (♂, MNHG) (Fig. 1D–F): “TAIWAN Taichung Hsien / Hseuhshan, nr. Hseuhshan- / Tun-Feng 3170 m 11.V.91 / A. Smetana [176] [p, w] // HOLOTYPE / Batophila / taiwanica / det. Döberl 1997 [p, r] // MHNG / ENTO / 0261580 [p, w]”.

#### Additional materials examined (n = 1346).

**Taiwan** • **Hsinchu**: 1♂ (NMNS), Ching Chuan (清泉), 21.XII.1989, leg. K. W. Huang; • 1♂, 1♀ (NMNS), Kuanwu (觀霧), 25–29.IV.1989, leg. C. S. Lin; • 4♀♀ (TARI), same locality, 30.IV.2010, leg. C.-F. Lee; • 2♀♀ (TARI), same but with “leg. M.-H. Tsou; • 1♂ (TARI), Peitelaman (北得拉曼), 26.VI.2008, leg. S.-F. Yu; • 2♀♀ (TARI), Tahunshan (大混山), 24.II.2009, leg. S.-F. Yu; • 1♀ (TARI), same but with “8.IX.2009”; • 4♀♀ (TARI), Talulintao (大鹿林道), 17.II.2008, leg. M.-H. Tsou; • 3♀♀ (TARI), Wufeng (五峰), 8.II.2009, leg. H.-J. Chen; • **Hualien**: 1♂ (TARI), Karenko (= Hualien, 花蓮), 20.VII.–4.VIII.1919, leg. T. Okuni, identified as *B. yangweii* by Chûjô (1937); • 2♂♂ (TARI), Kuanyuan (關原), 2.VI.2016, leg. B.-X. Guo; • 1♂ (TARI), same but with “leg. Y.-T. Chung”; • 2♂♂, 6♀♀ (KMNH), Tayuling (大禹嶺), 21.VI.1976, leg. H. Makihara; • 14♂♂, 11♀♀ (TARI), same locality, 9–16.VI.1980, leg. K. S. Lin & B. H. Chen; • 7♂♂, 11♀♀ (TARI), same locality and collectors, 10–16.VI.1980, Malaise trap; • 2♂♂, 5♀♀ (TARI), same locality, 12–15.IX.1980, leg. K. S. Lin & C. H. Wang; • 5♂♂, 1♀ (NMNS), same locality (= Tai Yu Lin), 25.VII.1990, leg. W. C. Chuang; • 1♀ (NHMUK), SW above Tayuling, 24°10'N, 121°17'30E, 2950 m, 31.X.2008, leg. L. Dembický; • **Ilan**: 3♂♂, 3♀♀ (TARI), Taipingshan (太平山), 8.VII.2008, leg. H.-J. Chen; • 1♂ (TARI), same locality, 19.II.2009, leg. H. Lee; • 1♂, 4♀♀ (TARI), same locality, 30.IV.2009, leg. C.-F. Lee; • 3♂♂, 3♀ (TARI), Tsuifenghu (翠峰湖), 3.VII.2010, leg. M.-H. Tsou; • **Miaoli**: 1♀ (TARI), Hsuehchien (雪見), 6.XII.2013, leg. W.-B. Yeh; • 3♂♂ (TARI), same but with “23.III.2014”; • **Nantou**: 1♂, 1♀ (KMNH), Hohuangshan (合歡山), 3.VI.1971, leg. Kanmiya; • 1♀ (NMNS), same locality (Ho Huang Shan), 26.VII.1990, leg. W. C. Chuang; • 5♀♀ (TARI), same locality (= Hehuanshan), 18.V.2009, leg. M.-H. Tsou; • 1♂ (TARI), Huakang (華岡), 26.VII.2010, leg. M.-H. Tsou; • 9♂♂, 27♀♀ (TARI), Meifeng (梅峰), 10.V.1979, leg. K. C. Chou; • 8♀ (TARI), same locality, 18.VII.1979, leg. K. C. Chou; • 12♂♂, 20♀♀ (TARI), same locality, 2–4.VI.1980, leg. L. Y. Chou & C. C. Chen; • 3♂♂, 19♀♀ (TARI), same locality, 8.VI.1980, leg. K. S. Lin & B. H. Chen; • 3♂♂, 12♀♀ (TARI), same locality, 26.VIII.1980, leg. K. S. Lin & C. H. Wang; • 1♀ (TARI), same locality, 5–9.X.1980, leg. C. C. Chen & C. C. Chien; • 13♂♂, 35♀♀ (TARI), same locality, 7–9.V.1981, leg. K. S. Lin & S. C. Lin; • 57♂♂, 59♀♀ (TARI), same locality, 24–26.VI.1981, leg. K. S. Lin & W. S. Tang; • 12♂♂, 16♀♀ (TARI), same locality, 28–29.VIII.1981, leg. L. Y. Chou & S. C. Lin; • 1♂, 1♀ (TARI), same locality, 7.XI.1981, leg. S. C. Lin & W. S. Tang; • 2♂♂, 2♀♀ (TARI), same locality, 22.V.1982, leg. L. Y. Chou; • 50♂♂, 62♀♀ (TARI), same locality, 15.VII.1982, S. C. Lin & C. N. Lin; • 17♂♂, 25♀♀ (TARI), same locality, 31.VIII.–2.IX.1982, leg. L. Y. Chou & K. C. Chou; • 23♂♂, 28♀♀ (TARI), same locality, 4–7.X.1982, leg. K. C. Chou; • 2♀♀ (TARI), same locality, 19–21.IV.1983, leg. K. C. Chou & S. P. Huang; • 4♂♂, 10♀♀ (TARI), same locality, 30.VII.1983, leg. L. Y. Chou; • 1♂, 3♀♀ (NMNS), same locality, 27.II.1992, leg. Y. C. Shiau; • 2♂♂ (NMNS), same locality, 14.XII.2004–11.I.2005, leg. C. S. Lin & W. T. Yang, Malaise trap; • 1♂, 1♀ (NMNS), same but with “11.I.–15.II.2005”; • 1♂ (NMNS), same but with “3.V.–7.VI.2005”; • 1♂, 2♀ (NMNS), same but with “9.I.–6.II.2007”; • 1♂, 3♀♀ (NMNS), same but with “6.II.–13.III.2007”; • 1♀ (NMNS), same but with “13.III.–10.IV.2007”; • 2♀♀ (KMNH), Piluchi (碧綠溪), 5.VIII.1986, leg. K. Baba, identified as *B. acutangula* by Kimoto (1989); • 5♂♂ (NMNS), same locality, 4.XII.1991, leg. Y. C. Shiau; • 1♂, 2♀♀ (NMNS), Piluhsi For. Res. Stn. (畢祿溪試驗站), 23–24.V.1999, leg. C. W. & L. B. O’Brien; • 1♂, 1♀ (TARI), Sungkang (松崗), 6.VIII.1984, leg. K. S. Lin; • 15♂♂, 43♀♀ (TARI), same locality, 15–17.VIII.1984, leg. K. C. Chou; • 19♂♂, 17♀♀ (TARI), same locality, 13–15.IX.1984, leg. K. S. Lin & S. C. Lin; • 2♂♂, 1♀ (NHMUK), (near Sungkang, 松崗) sheep farm, 24°03.121'N, 121°09.643'E, 1916 m, 7.VIII.2008, leg. M. V. L. Barclay & Mendel; • 3♂♂, 5♀♀ (KMNH), Sungkang (松崗) – Tsifen (sic!) (翠峰), 29.VI.1965, leg. S. Kimoto, identified as *B. acutangula* by Kimoto (1971); • 2♂♂, 2♀♀ (KMNH), Tsuifeng (翠峰), 26.VII.1971, leg. Y. Miyake; • 8♂♂, 10♀♀ (TARI), same locality, 21.VI.1979, leg. K. S. Lin & B. H. Chen; • 4♂♂, 16♀♀ (TARI), same locality, 3.VI.1980, leg. L. Y. Chou & C. C. Chen; • 77♂♂, 82♀♀ (TARI), same locality, 25–27.VI.1981, leg. K. S. Lin & W. S. Tang; • 11♂♂, 22♀♀ (TARI), same locality, 1–3.VIII.1981, leg. T. Lin & W. S. Tang; • 1♀ (TARI), same locality, 8.XI.1981, leg. S. C. Lin & W. S. Tang; • 5♂♂, 6♀♀ (TARI), same locality, 23.V.1982, leg. L. Y. Chou; • 11♂♂, 8♀♀ (TARI), same locality, 1–3.IX.1982, leg. L. Y. Chou & K. C. Chou; • 1♂, 1♀ (TARI), same locality, 6.X.1982, leg. K. C. Chou; • 1♂, 13♀♀ (TARI), same locality, 20.IV.1983, leg. K. C. Chou & S. P. Huang; • 2♂♂, 18♀♀ (TARI), same locality, IV.1984, Malaise trap, leg. K. S. Lin & K. C. Chou; • 59♂♂, 68♀♀ (TARI), same locality, 9.V.1984, leg. K. C. Chou & C. C. Pan; • 4♂♂, 21♀♀ (TARI), same locality, 5.VIII.1984, leg. K. S. Lin; • 2♂♂, 8♀♀ (TARI), same locality, 15–16.VIII.1984, leg. K. C. Chou; • 3♂♂, 7♀♀ (TARI), same locality, 12–14.IX.1984, leg. K. S. Lin & S. C. Lin; • 28♂♂, 30♀♀ (TARI), Tungpu (東埔), 20–22.VI.1980, leg. C. C. Chen; • 1♀ (TARI), same locality, 25–29.IX.1980, leg. L. Y. Chou & T. Lin; • 1♀ (TARI), same locality, 28.IV.–2.V.1981, leg. T. Lin & C. J. Lee; • 1♂, 1♀ (TARI), same locality, 18–23.XI.1981, leg. T. Lin & W. S. Tang; • 1♂ (TARI), same locality, 19–23.VII.1982, leg. L. Y. Chou & T. Lin; • 2♀♀ (TARI), same locality, 20–24.VI.1983, leg. K. C. Chou & C. Y. Wong; • 2♂♂, 2♀♀ (TARI), same locality, 16–20.IV.1984, leg. K. C. Chou & C. H. Yung; • 1♂ (NMNS), same locality, 31.I.1989, leg. K. W. Huang; • 1♂ (NMNS), Yuanfeng (鳶峰), 19.II.–12.III.2002, leg. C. S. Lin & W. T. Yang, Malaise trap; • **Taichung**: 2♂♂, 3♀♀ (NMNS), Anmashan (鞍馬山), 3.V.1992, leg. C. Y. Li; • 2♂♂, 2♀♀ (TARI), same locality 21.IV.2010, leg. C.-F. Lee; • 6♀♀ (TARI), Pilu (畢祿), 17.V.2009, leg. C.-F. Lee; • 1♂, 2♀♀ (TARI), Tahsuehshan (大雪山), 18.IV.2011, leg. J.-C. Chen; • **Taoyuan**: 1♂ (KMNH), Palon (巴陵?), 3.VIII.1986, leg. K. Baba, identified as *B. acutangula* by Kimoto (1989).

#### Diagnosis.

**Figure 20.**
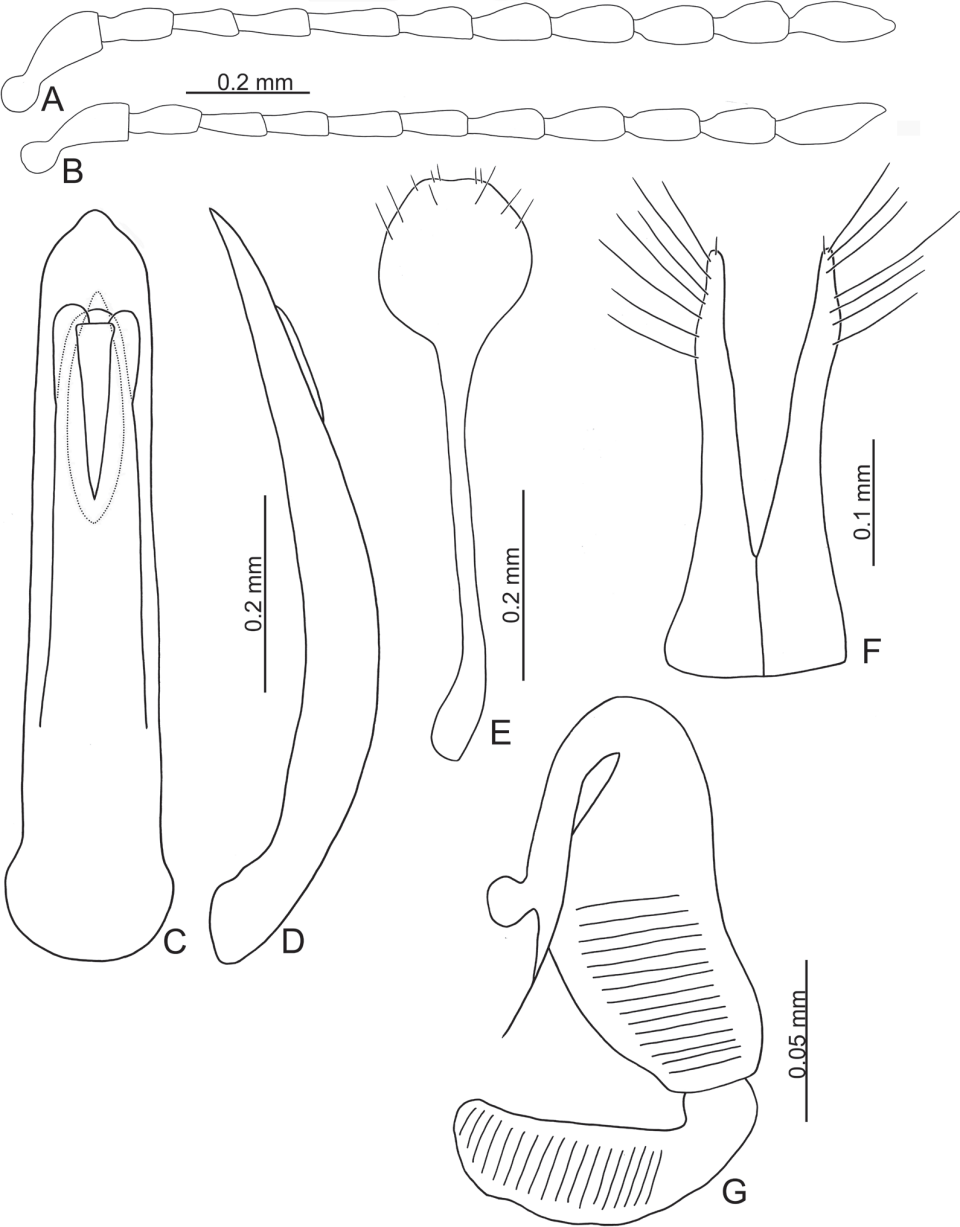
*Batophila taiwanica* Döberl A. Antenna, male; B. Antenna, female; C. Aedeagus, dorsal view; D. Aedeagus, lateral view; E. Abdominal ventrite VIII, female; F. Spermatheca; G. Gonocoxae.

Adults of *B. taiwanica* Döberl are similar to those of *B. yehi* sp. nov. and *B. meihuai* sp. nov. in possessing convergent elytral apices. However, adults of *B. taiwanica* (Fig. 20A, B) and *B. meihuai* sp. nov. (Fig. 18A, B) have slender antenna, length of antennomeres VI–X > 0.5× antennomere I [stout antenna in *B. yehi* sp. nov., antennomeres VI–X 0.5× antennomere I (Fig. 25A, B)]. Adults of *B. taiwanica* (Fig. 19) and *B. yehi* sp. nov. (Fig. 24) possess indistinct or reduced longitudinal ridges on the elytra which are different from *B. meihuai* sp. nov. by their distinct and sexually dimorphic longitudinal ridges on the elytra (Fig. 17). The aedeagus of these species are diagnostic: rounded apex with narrowly rounded process at middle of apical margin in *B. taiwanica* (Fig. 20C), widely rounded apex in *B. yehi* sp. nov. (Fig. 25C), and truncated apex in *B. meihuai* sp. nov. (Fig. 18C).

#### Description.

Male. Length 1.52–1.67 mm, width 0.80–0.83 mm. General color metallic dark bronze (Fig. 19A–C); antennae yellowish brown but six apical antennomeres darker; legs yellowish but apical halves of femora of hind legs darkened. Antenna (Fig. 20A) filiform and antennomeres VIII–X wide, ratio of length of antennomeres I–XI to length of antennomere I 1.0: 0.6: 0.5: 0.6: 0.7: 0.6: 0.7: 0.7: 0.7: 0.6: 0.9; ratio of length to width of antennomeres I–XI 2.7: 2.3: 2.4: 2.7: 3.2: 2.4: 2.2: 2.4: 2.0: 1.8: 2.7. Pronotum 1.21–1.23× wider than long; lateral margins slightly rounded, anterolateral angles separated from lateral margins by weak emarginations, slightly narrowed basally, distance between anterolateral angles 1.04–1.08× wider than basal margin. Elytra 1.35–1.36× longer than wide; lateral margins rounded, widest at basal 1/3, apex rounded but divergent; dorsoventrally flattened, apex visible in dorsal view; disc with longitudinal lines of coarse punctures and with distinct longitudinal grooves along lines. Tarsomeres I of front and middle legs slightly swollen. Aedeagus (Fig. 20C, D) elongate, 5.8× longer than wide; lateral margins basally and slightly widened towards base, apex narrowly rounded; dorsal opening starting from apical 1/7 and basally membranous, tectum composed of three lobes, median lobe more ventral relative to lateral lobes and apical margin truncate, mostly membranous; moderately curved in lateral view; ventral surface with membranous area narrower than dorsal opening, starting from apical 1/10–2/5.

Female (Fig. 19D–F). Length 1.96–2.04 mm, width 1.06–1.13 mm. Antennae similar to males, ratio of length of antennomeres I–XI to length of antennomere I (Fig. 20B) 1.0: 0.6: 0.6: 0.5: 0.7: 0.6: 0.7: 0.7: 0.7: 0.7: 1.0; ratio of length to width of antennomeres I–XI 2.8: 2.3: 2.6: 2.6: 3.0: 2.5: 2.5: 2.5: 2.4: 2.0: 3.1. Elytra 1.29–1.36× longer than wide; lateral margins rounded, widest at basal 1/3, apex rounded but divergent; dorsoventrally convex, apex not visible in dorsal view; disc with longitudinal lines of coarse punctures and with distinct longitudinal grooves along punctures. Gonocoxae (Fig. 20F) slender, connected at basal 1/5; each gonocoxa with seven long setae and one tiny seta from apical 1/5 to apex, subapically slightly curved. Ventrite VIII (Fig. 20E) weakly sclerotized apically, with several short setae at sides of apex, and some tiny setae at sides of apical margin, spiculum extremely elongate. Spermathecal receptaculum (Fig. 20G) strongly swollen, with transverse wrinkles at basal 1/2; pump wide and curved, with transverse wrinkles at apical 2/3; sclerotized spermathecal canal moderately long before base of spermathecal gland.

#### Food plants.

Rosaceae: *Rubus croceacanthus* H. Lév.

#### Distribution.

This species is widespread in mountainous areas of central Taiwan (Fig. 4).

### ﻿ Batophila tsoui sp. nov.

Taxon classificationAnimaliaColeopteraChrysomelidae

3461572B-B7C4-5F7B-966F-9F903DF32E10

https://zoobank.org/20C50E88-25E5-4D8D-81CA-70F628403ABD

Figs 21, 22

Batophila acutangula : Kimoto 1989: 269 (part).
Batophila yangweii : Chûjô 1937: 54 (part).

#### Type specimens examined (n = 178).

***Holotype*** ♂ (TARI): **Taiwan** • **Kaohsiung**: Chuyunshan logging trail (出雲山林道), 24.III.2009, leg. C.-F. Lee. ***Paratypes***. 3♂♂, 7♀♀ (TARI), same data as holotype; **Taiwan** • **Chiayi**: 1♂, 1♀ (TARI), Arisan (= Alishan, 阿里山), 10.X.1912, leg. I. Nitobe, both identified as *B. yangweii* by Chûjô (1937); • 1♀ (TARI), same locality, 2–23.X.1918, leg. J. Sonan, identified as *B. yangweii* by Chûjô (1937); • 1♀ (TARI), same locality, 10.VI.1940, leg. M. Chujo; • 1♂, 1♀ (KMNH), same locality, 9.IV.1965, leg. Y. Hirashima, the female identified as *B. acutangula* in 1975; • 1♂, 1♀ (KMNH), same locality, 17.V.1968, leg. B.-S. Chang, both identified as *B. acutangula* by Kimoto in 1973; • 1♀ (KMNH), same locality, 5.V.1971, leg. K. Kamiya; • 1♀ (KMNH), same but with “21.V.1971”; • 3♀♀ (KMNH), same but with “26.V.1971”, of which one is identified as *B. acutangula* by Kimoto in 1971 ; • 1♂ (KMNH), same locality, 22–25.VI.1974, leg. M. Owada; • 7♂♂, 4♀♀ (TARI), same locality, 5–9.VIII.1981, leg. L. Y. Chou & S. C. Lin; • 7♂♂, 4♀♀ (TARI), same locality, 17–20.VIII.1982, leg. K. C. Chou & C. C. Pan; • 1♀ (NMNS), Fenchifu (奮起湖), 14.XII.1988, leg. K. W. Huang; • **Kaohsiung**: 1♀ (TARI), Erchituan (二集團), 8.III.2013, leg. B.-X. Guo; • 1♂ (TARI), Shihshan (石山), 30.XII.2008–6.I.2009, leg. C.-T. Yao; • 4♂♂, 4♀♀ (KMNH), same locality (= Shyk Shan), 25.IV.1986, leg. K. Baba, identified as *B. acutangula* by Kimoto (1989); • 3♀♀ (TARI), Shihshan logging trail (石山林道), 24.III.2009, leg. M.-H. Tsou; • 10♂♂, 4♀♀ (NMNS), Tengchih (藤枝), 7.IX.1989, leg. K. W. Huang; • 1♀ (NMNS), same locality, 21–24.XI.1995, leg. M. L. Chan; • 1♂ (TARI), same locality, 18.II.2007, leg. S.-F. Yu; • 1♂ (TARI), same locality, 2–5.VI.2008, leg. C.-F. Lee; • 4♀♀ (TARI), same locality, 9.XI.2013, leg W.-C. Liao; • 1♀ (KMNH), Tienchi (天池), 2.VI.1986, leg. K. Baba, identified as *B. acutangula* by Kimoto (1989); • 1♂, 5♀♀ (NMNS), Tona forest road (多納林道), 28.IV.1998, leg. M. L. Chan; • 1♀ (TARI), same locality (= Tonalintao), 16.II.2011, leg. J.-C. Chen; • 2♀♀ (KMNH), Yakou (啞口), 1.VIII.1986, leg. K. Baba, identified as *B. acutangula* by Kimoto (1989); • **Nantou**: 10♂♂, 15♀♀ (TARI), Hsitou (溪頭), 25.IV.2025, leg. C.-F. Lee; • 1♂ (TARI), Tatachia (塔塔加), 29.X.2009, leg. C.-F. Lee; • 1♂ (TARI), same but with “9.V.2011”; • 5♂♂, 8♀♀ (TARI), same but with “23.IV.2025”; • **Pingtung**: 2♂♂, 5♀♀ (TARI), Peitawushan (北大武山), 13.III.2025, leg. J.-C. Chen; • 2♂♂, 3♀♀ (TARI), same but with “20.III.2025”; • **Taitung**: 2♂♂, 3♀♀ (MHNUK), 14 km W of Chihshang (池上), 23°09'N, 121°04'E, 900 m, 16.XI.2008, leg. L. Dembický; • 1♂, 6♀♀ (TARI), Hsiangyang (向陽), 1.VII.2009, leg. M.-H. Tsou; • 3♂♂, 4♀♀ (TARI), Motien (摩天), 23.V.2011, leg. C.-F. Lee; • 1♂, 1♀ (TARI), Liyuan (栗園), 24.I.2014, leg. W.-C. Huang; • 2♂♂, 1♀ (TARI), same but with “14.III.2014”; • 1♀ (NHMUK), Yakou Country Inn (啞口山莊), 23°16.063'N, 120°58.419'E, 2582 m, 11.VIII.2008, leg. H. Mendel & M. V. L. Barclay; • **Yunlin**: 3♂♂, 1♀ (NMNS), Shihpi (石壁), 20.II.1991, leg. C. C. Chiang; • 1♂, 1♂ (NMNS), same but with “22.II.1991”; • 2♂♂, 1♀ (NMNS), same locality, 27.X.1992, leg. W. T. Yang.

#### Diagnosis.

**Figure 21.**
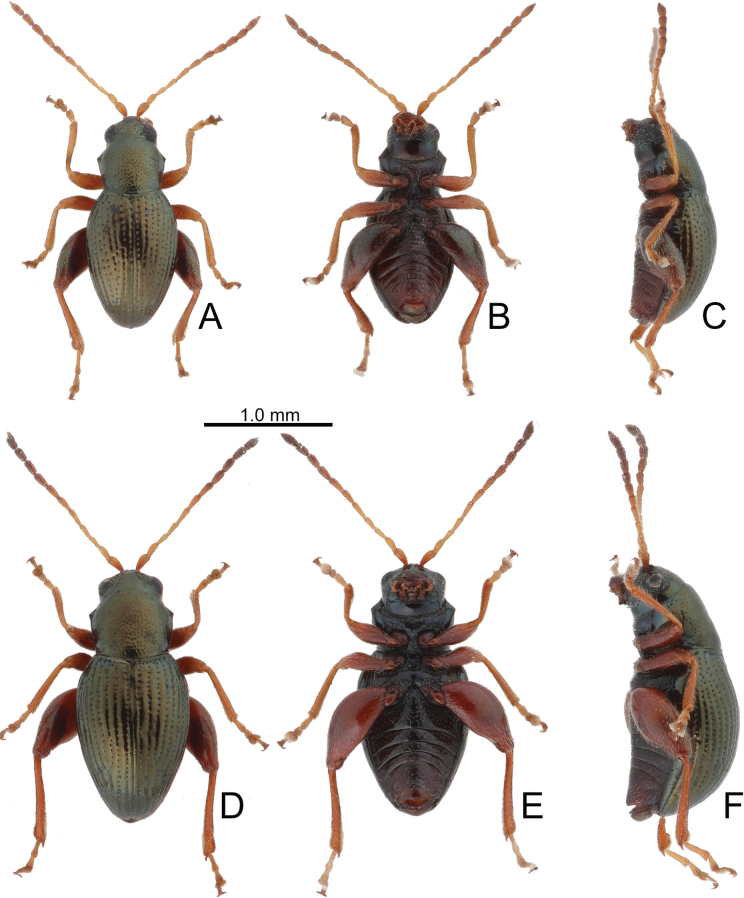
Habitus of *Batophila tsoui* sp. nov. A. Male, paratype, dorsal view; B. Ditto, ventral view; C. Ditto, lateral view; D. Female, paratype, dorsal view; E. Ditto, ventral view; F. Ditto, lateral view.

**Figure 22.**
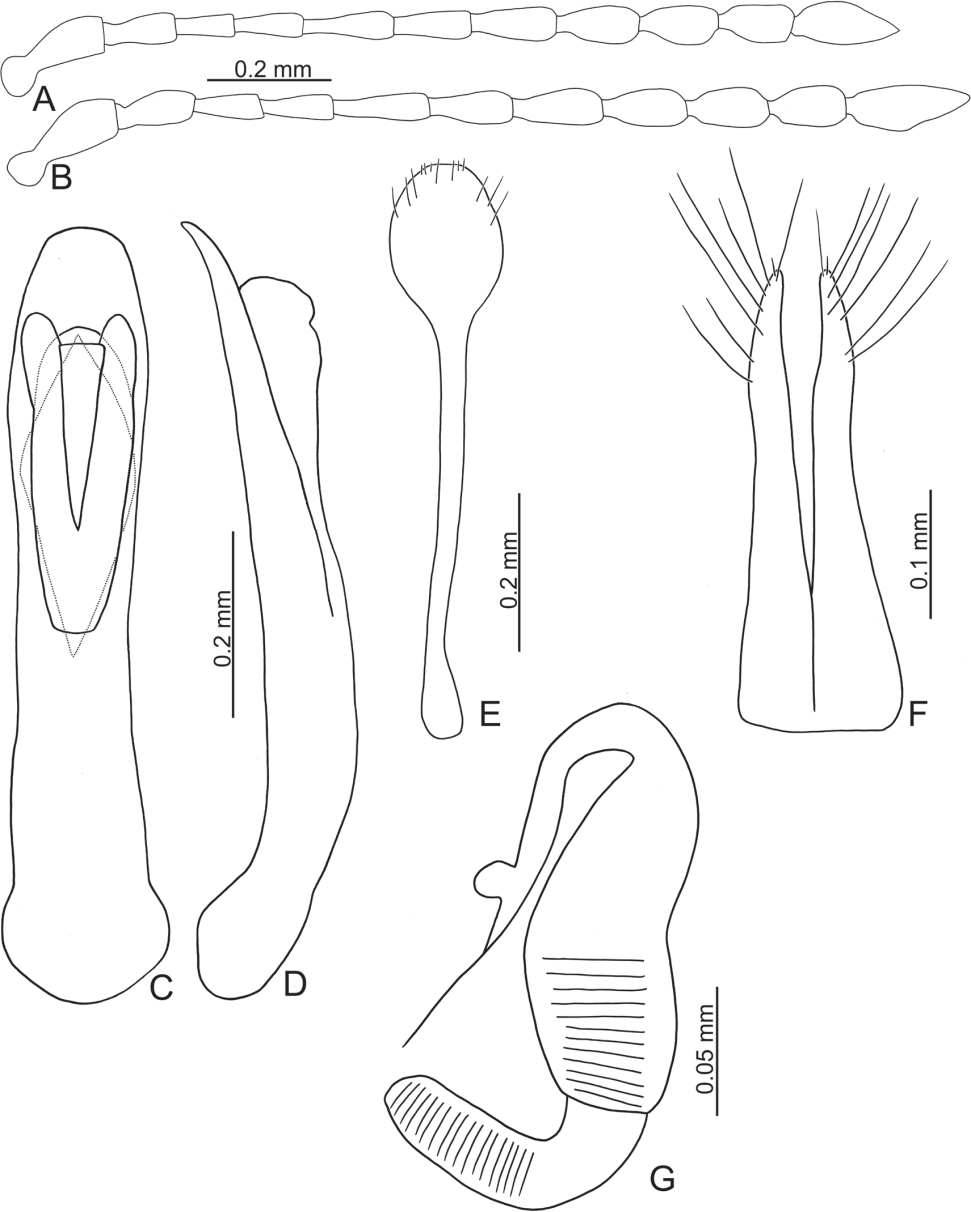
*Batophila tsoui* sp. nov. A. Antenna, male; B. Antenna, female; C. Aedeagus, dorsal view; D. Aedeagus, lateral view; E. Abdominal ventrite VIII, female; F. Spermatheca; G. Gonocoxae.

Adults of *B. tsoui* sp. nov., *B. chungi* sp. nov., and *B. choui* sp. nov. are recognized by their strongly and apically narrowed elytra, and divergent elytral apices, but *B. tsoui* sp. nov. and *B. chungi* sp. nov. differ in possessing flattened elytra in males (Fig. 21C) but convex elytra in females (Fig. 21F) [convex elytra and elytral apex not visible in dorsal views in both sexes of *B. choui* sp. nov. (Fig. 5C, F)], and widened apex of aedeagus (Fig. 22C) [parallel-sided aedeagus in *B. choui* sp. nov. (Fig. 6C)]. Adults of *B. tsoui* sp. nov. are not separable from those *B. chungi* sp. nov. by external morphology but the aedeagus of *B. tsoui* sp. nov. (Fig. 22C) is narrower than that of *B. chungi* sp. nov. (Fig. 8C).

#### Description.

Male. Length 1.86–1.94 mm, width 0.77–0.80 mm. General color metallic dark bronze (Fig. 21A–C); legs yellowish but femora of hind legs darkened. Antenna (Fig. 22A) filiform and antennomeres VIII–X wide, ratio of length of antennomeres I–XI to length of antennomere I 1.0: 0.7: 0.5: 0.6: 0.8: 0.7: 0.8: 0.7: 0.7: 0.7: 0.9; ratio of length to width of antennomeres I–XI 2.8: 2.5: 2.4: 3.0: 3.3: 2.6: 2.4: 2.3: 2.0: 2.1: 2.5. Pronotum 1.17–1.22× wider than long; lateral margins slightly rounded, anterolateral angles separated from lateral margins by weak emarginations, slightly narrowed basally, distance between anterolateral angles 1.14–1.22× wider than basal margin. Elytra 1.48–1.49× longer than wide; lateral margins rounded, widest at basal 1/5, apically and strongly narrowed, apex truncate but divergent; dorsoventrally flattened, apex visible in dorsal view; disc with longitudinal lines of extremely coarse punctures and with distinct longitudinal grooves along punctures, punctures and grooves apically abbreviated from apical 1/3. Tarsomeres I of front and middle legs slightly swollen. Aedeagus (Fig. 22C, D) elongate, 5.8× longer than wide; widest at apical 1/5, apically narrowed towards apex, apex widely rounded, basally widened near apical 2/5, then widened near base; dorsal opening starting from apical 1/9–1/3, tectum composed of three lobes, median lobe more ventral relative to lateral lobes, apical margin truncate, mostly membranous; slightly curved in lateral view, apex moderately curved; ventral surface with membranous area wider than dorsal opening, starting from apical 1/10–3/5.

Female (Fig. 21D–F). Length 1.99–2.56 mm, width 0.88–1.05 mm. Antennae similar to males, ratio of length of antennomeres I–XI to length of antennomere I (Fig. 22B) 1.0: 0.6: 0.6: 0.6: 0.8: 0.7: 0.8: 0.7: 0.7: 0.6: 1.0; ratio of length to width of antennomeres I–XI 2.6: 2.3: 2.7: 3.0: 3.5: 2.6: 2.5: 2.2: 2.0: 1.9: 2.8. Elytra 1.46–1.67× longer than wide; lateral margins rounded, widest at basal 1/5, apex truncate but divergent; dorsoventrally convex, apex not visible in dorsal view; disc with longitudinal lines of extremely coarse punctures and with distinct longitudinal grooves along punctures, punctures and grooves apically abbreviated from apical 1/3. Gonocoxae (Fig. 22F) slender, connected at basal 1/5; each gonocoxa with seven long setae and one tiny seta from apical 1/5 to apex, subapically slightly curved. Ventrite VIII (Fig. 22E) weakly sclerotized apically, with several short setae at apical area, and some tiny setae at apical margin, spiculum extremely elongate. Spermathecal receptaculum (Fig. 22G) strongly swollen, with transverse wrinkles at basal 1/2; pump wide and curved, with transverse wrinkles at apical 2/3; sclerotized spermathecal canal moderately long before base of spermathecal gland.

#### Food plants.

Melastomataceae: *Otanthera scaberrima* (Hayata) Ohwi (Fig. 9E, F); Rosaceae: *Rubus formosensis* Kuntze, R. morii Hayata, R. croceacanthus H. Lév., *R. wallichianus* Wight & Arn. (Fig. 9D).

#### Etymology.

This new species is named for Mei-Hua Tsou (曹美華), the first member of TCRT to collect specimens.

#### Distribution.

This species is widespread in mountainous areas in southern Taiwan (Fig. 7).

### ﻿ Batophila wusheensis sp. nov.

Taxon classificationAnimaliaColeopteraChrysomelidae

713818CA-2B57-5CAF-850E-8074F52C3B89

https://zoobank.org/D26AAC6F-F64D-4394-8C84-85585A9F2AC6

Fig. 23

Batophila yangweii : Chûjô 1937: 54 (part).

#### Type specimens examined (n = 201).

***Holotype*** ♂ (TARI): **Taiwan. Nantou**: **Nantou**: Wushe (霧社), 30.VIII.–2.IX.1982, leg. L. Y. Chou & K. C. Chou. ***Paratypes***. 88♂♂, 55♀♀ (TARI), same data as holotype; **Nantou**: 5♂♂, 5♀♀ (NHMUK), (Chingying, 精英), 24°02.530'N, 121°12.555'N, 1920 m, 6.VIII.2008, leg. M. V. L. Barclay, H. Mendel & R. Ewers; 3♀♀ (NMNS), Chunyang (春陽), 9.IV.–7.V.2002, leg. C. S. Lin & W. T. Yang, Malaise trap; 1♂, 1♀ (TARI), Hoshe (和社), 22.VII.1982, leg. L. Y. Chou & T. Lin; 2♀♀ (TARI), Musha (= Wushe, 霧社), 18.V.–15.VI.1919, leg. T. Okuni, both identified as *B. yangweii* by Chûjô (1937); 1♂ (TARI), same locality, 23–28.VI.1981, leg. K. S. Lin & W. S. Tang; 1♂ (TARI), same locality, 26–28.VIII.1981, leg. L. Y. Chou & S. C. Lin; 1♀ (TARI), same locality, 14.VII.1982, leg. S. C. Lin & C. N. Lin; 9♂♂, 7♀♀ (TARI), same locality, 7–8.X.1982, leg. K. C. Chou; 14♂♂, 13♀♀ (TARI), same locality, 19–22.IV.1983, leg. K. C. Chou & S. P. Huang; 2♀♀ (NMNS), same locality, 29–31.V.1996, leg. C. S. Lin; 1♂, 1♀ (TARI), Yu-shih (幼獅), 4.VIII.1981, leg. T. Lin & W. S. Tang.

#### Diagnosis.

**Figure 23.**
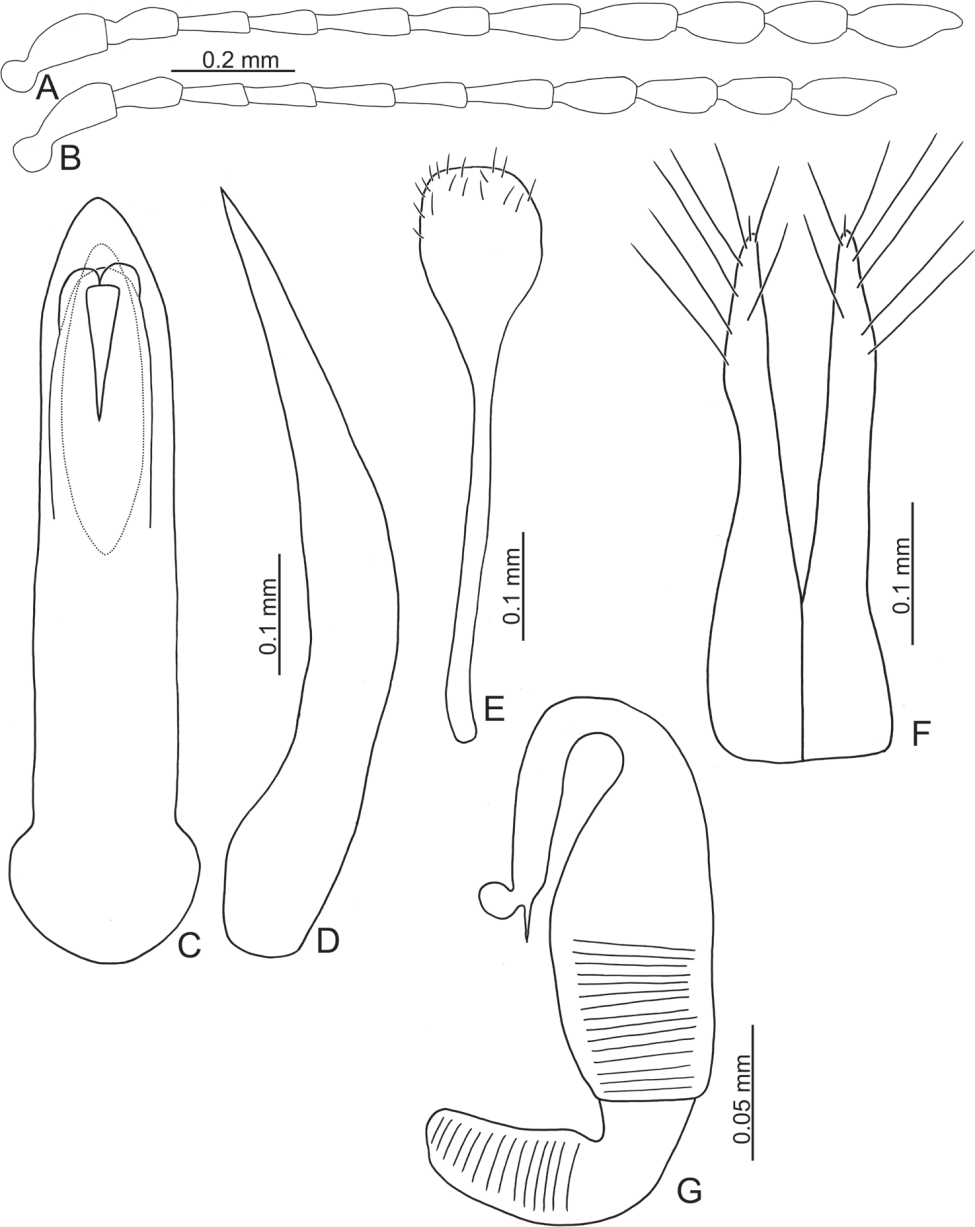
*Batophila wusheensis* sp. nov. A. Antenna, male; B. Antenna, female; C. Aedeagus, dorsal view; D. Aedeagus, lateral view; E. Abdominal ventrite VIII, female; F. Spermatheca; G. Gonocoxae.

Adults of *B. wusheensis* sp. nov. are not separable from those of *B. houjayi* sp. nov., *B. yuae* sp. nov., *B. jungchani* sp. nov., and *B. huangi* sp. nov. that are characterized by truncate elytral apices based on external morphology (Figs 10, 12) except for the aedeagus (see below). However, these species can be recognized by their allopatric distributions [*B. wusheensis* sp. nov. inhabits at lowlands in Nantou County, *B. houjayi* sp. nov. in high mountains in Chiayi, Ilan, Hualien, and Nantou counties, *B. yuae* sp. nov. in lowlands in Taipei and New Taipei Cities, and Ilan County, *B. jungchani* sp. nov. in high mountains in Taichung and Miaoli counties, *B. huangi* sp. nov. in lowlands in Miaoli County and high mountains in Hsinchu and Taoyuan counties (Fig. 13)]. Aedeagal shapes are diagnostic [apically tapering aedeagus from apical 1/5 in *B. wusheensis* sp. nov. (Fig. 23), widely rounded apex of aedeagus in *B. houjayi* sp. nov. (Fig. 11C), rounded apex of aedeagus with truncate process at middle of apical margin in *B. yuae* sp. nov. (Fig. 26C), subapically tapering apex of aedeagus in *B. jungchani* sp. nov. (Fig. 15C), and rounded apex of aedeagus with small, rounded process at middle of apical margin in *B. huangi* sp. nov. (Fig. 14C)].

#### Description.

Male. Length 1.57–1.74 mm, width 0.70–0.80 mm. General color metallic dark bronze; antennae yellowish brown but six apical antennomeres darker; legs yellowish but femora of hind legs darkened. Antenna (Fig. 23A) filiform and antennomeres VIII–X wide, ratio of length of antennomeres I–XI to length of antennomere I 1.0: 0.6: 0.6: 0.6: 0.8: 0.7: 0.8: 0.8: 0.8: 0.7: 1.0; ratio of length to width of antennomeres I–XI 2.6: 2.3: 3.1: 2.7: 3.5: 2.7: 2.5: 2.6: 2.1: 2.0: 2.7. Pronotum 1.22–1.24× wider than long; lateral margins slightly rounded, disc with lateral depressions at basal 1/3, anterolateral angles separated from lateral margins by weak emarginations, slightly narrowed basally, distance between anterolateral angles 1.09–1.17× wider than basal margin. Elytra 1.40–1.46× longer than wide; lateral margins rounded, widest at basal 1/3, apex truncate; dorsoventrally convex, apex not visible in dorsal view; disc with longitudinal lines of coarse punctures and with indistinct longitudinal grooves along lines, reduced in some individuals. Tarsomeres I of front and middle legs slightly swollen. Aedeagus (Fig. 23C, D) elongate, 5.8× longer than wide; parallel-sided, apically narrowed from apical 1/5, apex pointed; dorsal opening starting from apical 1/10 and basally membranous, tectum composed of three lobes, median lobe more ventral relative to lateral lobes and apical margin truncate, mostly membranous; moderately curved in lateral view; ventral surface with membranous area narrower than dorsal opening, starting from apical 1/20–1/2.

Female. Length 1.96–2.30 mm, width 0.87–1.00 mm. Antennae similar to males, ratio of length of antennomeres I–XI to length of antennomere I (Fig. 23B) 1.0: 0.6: 0.6: 0.6: 0.7: 0.6: 0.7: 0.7: 0.7: 0.6: 0.9; ratio of length to width of antennomeres I–XI 2.9: 2.2: 2.7: 2.7: 3.5: 2.8: 2.9: 2.4: 2.2: 2.1: 2.8. Elytra 1.42–1.51× longer than wide; lateral margins rounded, widest at basal 1/3, apex truncate; dorsoventrally convex elytral apex not visible in dorsal view; disc with longitudinal lines of coarse punctures and with indistinct longitudinal grooves along lines, reduced in some individuals. Gonocoxae (Fig. 23F) slender, connected at basal 1/5; each gonocoxa with seven long setae and one tiny seta from apical 1/5 to apex, subapically slightly curved. Ventrite VIII (Fig. 23E) weakly sclerotized apically, with several short setae at apical area, and some tiny setae at sides of apical margin, spiculum extremely elongate. Spermathecal receptaculum (Fig. 23G) strongly swollen, with transverse wrinkles at basal 1/2; pump wide and curved, with transverse wrinkles at apical 2/3; sclerotized spermathecal canal moderately long before base of spermathecal gland.

#### Food plants.

Unknown.

#### Etymology.

This new species is named after its type locality, Wushe (霧社).

#### Distribution.

Only known from the abovementioned localities in central Taiwan (Fig. 13).

### ﻿ Batophila yehi sp. nov.

Taxon classificationAnimaliaColeopteraChrysomelidae

07D72C33-5272-51A2-8DA9-AF1ACA2AD637

https://zoobank.org/690CABBE-A769-46DD-B9D5-D8F034D57B7B

Figs 24, 25

#### Type specimens examined (n = 99).

***Holotype*** ♂ (TARI). **Taiwan** • **Taichung**: Hsuehshan (雪山), 18.VI.2010, leg. W.-B. Yeh. ***Paratypes*** • 18♂♂, 12♀♀ (TARI), same data as holotype; • 15♂♂, 6♀♀ (TARI), same but with “3.V.2007”; • 1♀ (TARI), same but with “14.VI.2008”; • 5♂♂, 1♀ (TARI), same but with “4.VIII.2010”; • 1♀ (TARI), same but with “8.IV.2011”; • 12♂♂, 8♀♀ (TARI), same but with “10.VI.2011”; • 1♂ (TARI), same but with “3.VIII.1911”; • 1♀ (TARI), same locality, 29.IV.-28.VI.2012, leg. L.-P. Hsu; • **Miaoli**: 1♀ (TARI), Hsuehchien (雪見), 23.III.2014, leg. W.-B. Yeh; • **Nantou**: 6♂♂, 10♀♀ (TARI), Hehuanshan (合歡山), 23.VI.2018, leg. H.-F. Lu.

#### Diagnosis.

**Figure 24.**
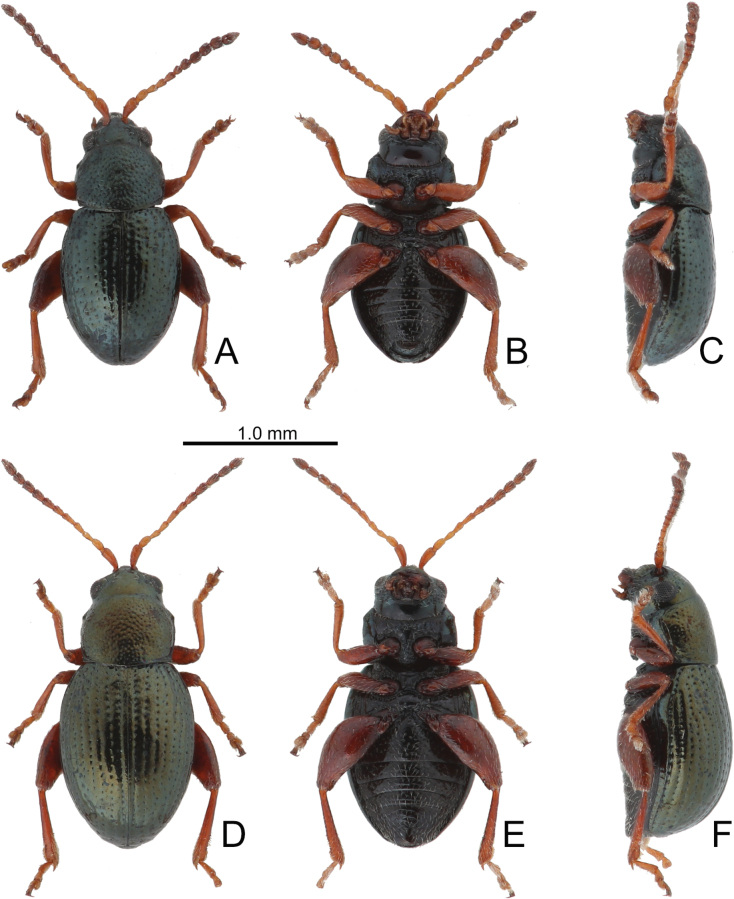
Habitus of *Batophila yehi* sp. nov. A. Male, paratype, from Hsuehshan (雪山), dorsal view; B. Ditto, ventral view; C. Ditto, lateral view; D. Female, paratype, from Hehuanshan (合歡山), dorsal view; E. Ditto, ventral view; F. Ditto, lateral view.

**Figure 25.**
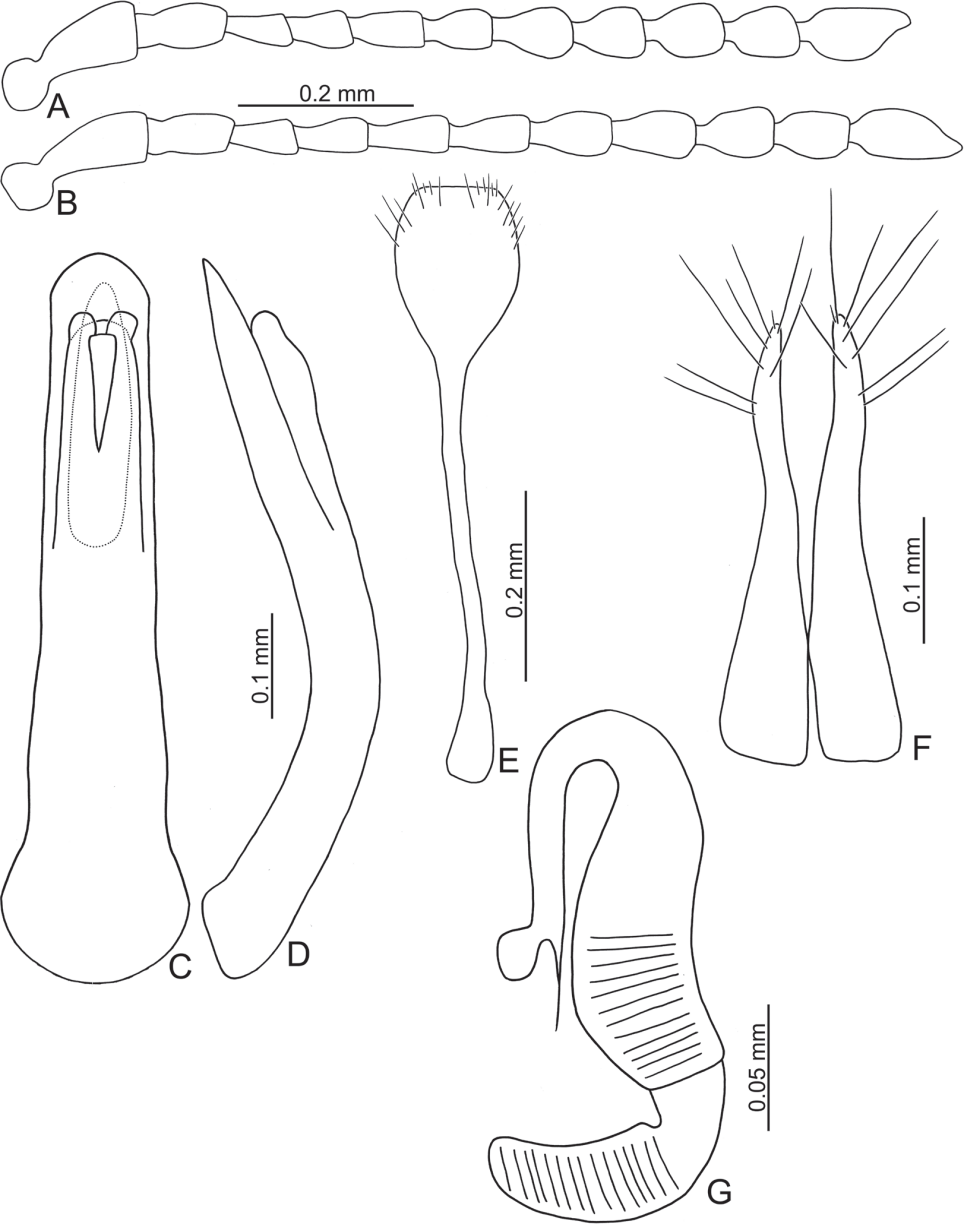
*Batophila yehi* sp. nov. A. Antenna, male; B. Antenna, female; C. Aedeagus, dorsal view; D. Aedeagus, lateral view; E. Abdominal ventrite VIII, female; F. Spermatheca; G. Gonocoxae.

Adults of *B. yehi* sp. nov. are similar to those of *B. taiwanica* Döberl and *B. meihuai* sp. nov. in possessing convergent elytral apices. However, adults of *B. yehi* sp. nov. are recognized by their stout antennae, length of antennomeres VI–X 0.5× length of antennomere I (Fig. 25A, B) [> 0.5× in *B. taiwanica* (Fig. 20A, B) and *B. meihuai* sp. nov. (Fig. 18A, B)]. Adults of *B. meihuai* sp. nov. are characterized by their distinct and sexually dimorphic longitudinal ridges on the elytra (Fig. 17) [indistinct or reduced longitudinal ridges on the elytra in *B. taiwanica* (Fig. 19) and *B. yehi* sp. nov. (Fig. 24)]. The aedeagi of these species are diagnostic: truncate apex in *B. meihuai* sp. nov. (Fig. 18C), rounded apex with narrowly rounded process at middle of apical margin in *B. taiwanica* (Fig. 20C), and widely rounded apex in *B. yehi* sp. nov. (Fig. 25C).

#### Description.

Male. Length 1.39–1.57 mm, width 0.69–0.78 mm. General color metallic dark bronze (Fig. 24A–C); antennae yellowish brown but six apical antennomeres darker; legs yellowish but femora of hind legs darkened. Antenna (Fig. 25A) filiform and antennomeres VIII–X wide, ratio of length of antennomeres I–XI to length of antennomere I 1.0: 0.5: 0.4: 0.5: 0.5: 0.5: 0.5: 0.5: 0.5: 0.5: 0.7; ratio of length to width of antennomeres I–XI 2.9: 2.0: 1.8: 2.0: 2.3: 2.2: 2.0: 1.9: 1.5: 1.6: 2.3. Pronotum 1.22–1.25× wider than long; lateral margins slightly rounded, anterolateral angles separated from lateral margins by weak emarginations, widest at middle, slightly narrowed basally, distance between anterolateral angles 0.99–1.01× wider than basal margin. Elytra 1.28–1.33× longer than wide; lateral margins rounded, widest at basal 1/3, apex widely rounded and converge; dorsoventrally flattened, apex visible in dorsal view; disc with longitudinal lines of coarse punctures but lacking longitudinal grooves and ridges. Tarsomeres I of front and middle legs strongly swollen. Aedeagus (Fig. 25C, D) elongate, 5.3× longer than wide; lateral margins basally and slightly widened towards base, apex widely rounded; dorsal opening starting from apical 1/10 and basally membranous, tectum composed of three lobes, median lobe more ventral relative to lateral lobes and apical margin truncate, mostly membranous; moderately curved in lateral view; ventral surface with membranous area narrower than dorsal opening, starting from apical 1/20–2/5.

Female. Length 1.63–1.73 mm, width 0.81–0.89 mm. Antennae similar to males, but antennomeres VIII–X wider than those of males, ratio of length of antennomeres I–XI to length of antennomere I (Fig. 25B) 1.0: 0.6: 0.4: 0.4: 0.5: 0.5: 0.5: 0.5: 0.5: 0.5: 0.8; ratio of length to width of antennomeres I–XI 2.4: 2.0: 1.9: 1.7: 2.0: 1.8: 1.5: 1.3: 1.4: 1.4: 2.1. Elytra 1.28–1.32× longer than wide; lateral margins rounded, widest at basal 1/3, apex convergent; dorsoventrally convex, apex not visible in dorsal view; disc with longitudinal lines of coarse punctures but lacking longitudinal grooves and ridges. Gonocoxae (Fig. 25F) slender, connected at basal 1/5; each gonocoxa with seven long setae and one tiny setae from apical 1/5 to apex, subapically slightly curved. Ventrite VIII (Fig. 25E) weakly sclerotized apically, with several short setae at sides of apex, and some tiny setae at sides of apical margin, spiculum extremely elongate. Spermathecal receptaculum (Fig. 25G) strongly swollen, with transverse wrinkles at basal 1/2; pump wide and curved, with transverse wrinkles at apical 2/3; sclerotized spermathecal canal moderately long before base of spermathecal gland.

#### Variation.

Individuals collected from Hehuanshan (合歡山) (Fig. 24D–F) have longitudinal grooves connected with lines of coarse punctures on the elytra.

#### Food plants.

Rosaceae: *Fragaria hayatai* Makino.

#### Etymology.

This new species is named for Dr. Wen-Bin Yeh (葉文斌), who worked as professor at the National Chung Hsing University and collected most of the type series.

#### Distribution.

This species is found in alpine areas of central Taiwan (Fig. 13).

### ﻿ Batophila yuae sp. nov.

Taxon classificationAnimaliaColeopteraChrysomelidae

5DFD2583-0143-544C-AF10-EAD69149DE9C

https://zoobank.org/F0E8D7D7-5893-4A66-B1C8-7CDA46803066

Fig. 26

Batophila yangweii : Chûjô 1937: 54 (part).

#### Type specimens examined (n = 20).

***Holotype*** ♂ (TARI). **Taiwan** • **Taipei**: Lengshuikeng (冷水坑), 29.VI.2008, leg. M.-H. Tsao (sic!). ***Paratypes*** • 2♀ (TARI), same data as holotype; • 1♀ (TARI), same locality, 22.II.2015, leg. M.-H. Tsou; • **Ilan**: 1♀ (NMNS), Chilan (棲蘭), 15.V.1999, leg. S. Halbert, C. W. & L. B. O’Brien; • 1♂ (TARI), Fushan Botanical Park (福山植物園), 19–26.VI.2006, leg. C.-S. Tung; • 1♀ (TARI), same but with “14–21.VII.2007”; • 2♂♂, 2♀♀ (TARI), Mingchi (明池), 5.IV.2009, leg. M.-H. Tsou; • 1♀ (TARI), Nishimura (= Hsitsun, 西村), 24.VII.1919, leg. Y. Miwa, identified as *B. yangweii* by Chûjô (1937); • 2♂ (NMNS), Wufangchi (sic!) (五峰旗), 16.X.1990, leg. C. C. Chiang; • **New Taipei City**: 1♂, 1♀ (TARI), Hinokiyama (= Kueishan, 檜山), 22.VII.1929, leg. Y. Miwa, both identified as *B. yangweii* by Chûjô (1937); • 1♀ (TARI), Wulai (烏來), 26.X.2006, leg. S.-F. Yu; • 1♀ (TARI), Yingtzuling (鶯子嶺), 9.V.2010, leg. M.-H. Tsou; • **Taipei City**: 1♀ (TARI), Sôzan (= Yangmingshan, 陽明山), 28.IV.1940, leg. S. Miyamoto; • 1♀ (TARI), Tatunshan (大屯山), 26.V.2010, leg. S.-F. Yu.

#### Diagnosis.

**Figure 26.**
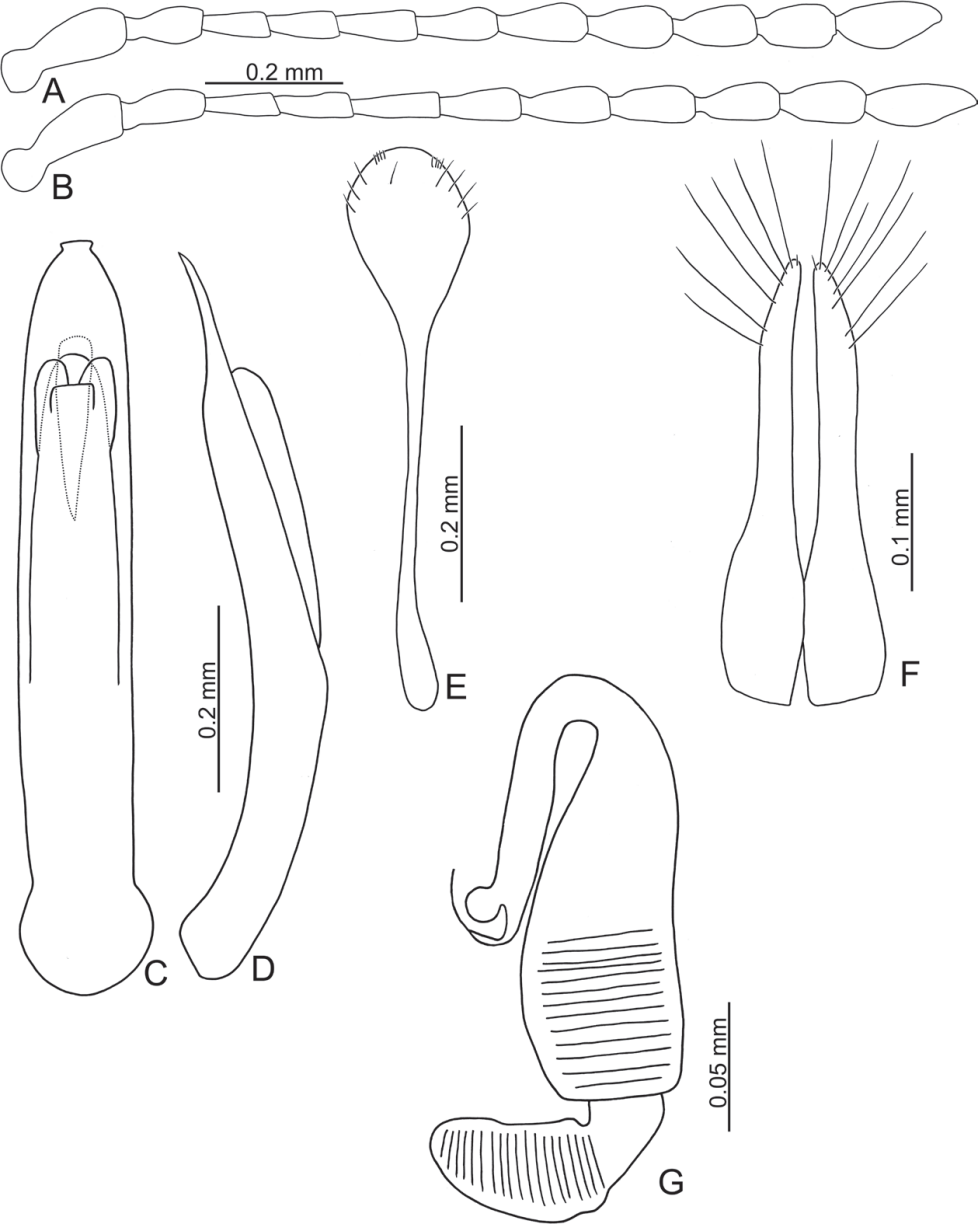
*Batophila yuae* sp. nov. A. Antenna, male; B. Antenna, female; C. Aedeagus, dorsal view; D. Aedeagus, lateral view; E. Abdominal ventrite VIII, female; F. Spermatheca; G. Gonocoxae.

Adults of *B. yuae* sp. nov. are not separable from those of *B. houjayi* sp. nov., *B. wusheensis* sp. nov., *B. jungchani* sp. nov., and *B. huangi* sp. nov. that are characterized by truncate elytral apices based on external morphology (Figs 10, 12) except for the aedeagus (see below). However, these species can be recognized by their allopatric distributions [*B. yuae* sp. nov. inhabits lowlands in Taipei and New Taipei Cities, and Ilan County, *B. wusheensis* sp. nov. in lowlands of Nantou County, *B. houjayi* sp. nov. in high mountains of Chiayi, Ilan, Hualien, and Nantou counties, *B. jungchani* sp. nov. in high mountains of Taichung and Miaoli counties, *B. huangi* sp. nov. in lowlands of Miaoli County and high mountains of Hsinchu and Taoyuan counties (Fig. 13)]. Aedeagal shapes are diagnostic [rounded apex of aedeagus with truncate process at middle of apical margin in *B. yuae* sp. nov. (Fig. 26C), apically tapering aedeagus from apical 1/5 in *B. wusheensis* sp. nov. (Fig. 23C), widely rounded apex of aedeagus in *B. houjayi* sp. nov. (Fig. 11C), subapically tapering apex of aedeagus in *B. jungchani* sp. nov. (Fig. 15C), and rounded apex of aedeagus with a small, rounded process at middle of apical margin in *B. huangi* sp. nov. (Fig. 14C)].

#### Description.

Male. Length 1.53–1.67 mm, width 0.76–0.80 mm. General color metallic dark bronze; antennae yellowish brown but six apical antennomeres darker; legs yellowish but femora of hind legs darkened. Antenna (Fig. 26A) filiform and antennomeres VIII–X wide, ratio of length of antennomeres I–XI to length of antennomere I 1.0: 0.6: 0.5: 0.5: 0.6: 0.6: 0.7: 0.7: 0.7: 0.6: 0.8; ratio of length to width of antennomeres I–XI 2.6: 2.1: 2.2: 2.4: 2.6: 2.5: 2.3: 2.0: 2.1: 1.8: 2.4. Pronotum 1.24–1.25× wider than long; lateral margins slightly rounded, anterolateral angles separated from lateral margins by weak emarginations, slightly narrowed basally, distance between anterolateral angles widest, 1.10–1.15× wider than basal margin. Elytra 1.31–1.34× longer than wide; lateral margins rounded, widest at basal 1/5, apex truncate; dorsoventrally flattened, apex visible in dorsal view; disc with longitudinal lines of coarse punctures, and indistinct longitudinal grooves along punctures present only near base, no ridges present between longitudinal grooves. Tarsomeres I of front and middle legs slightly swollen. Aedeagus (Fig. 26C, D) elongate, 6.6× longer than wide; parallel-sided, apically and strongly tapering near apex, apex with one transverse process, apical margin truncate; dorsal opening starting from apical 1/7 and basally membranous, tectum composed of three lobes, median lobe more ventral relative to lateral lobes and apical margin truncate, mostly membranous; moderately curved in lateral view; ventral surface with membranous area slender and small, starting from apical 1/8–1/3.

Female. Length 1.74–1.78 mm, width 0.86–0.92 mm. Antennae similar to males, ratio of length of antennomeres I–XI to length of antennomere I (Fig. 26B) 1.0: 0.6: 0.5: 0.6: 0.7: 0.6: 0.7: 0.6: 0.6: 0.6: 0.8; ratio of length to width of antennomeres I–XI 2.8: 2.5: 2.9: 2.9: 3.4: 2.5: 2.5: 2.3: 2.2: 2.0: 2.5. Elytra 1.31–1.38× longer than wide; lateral margins rounded, widest at basal 1/3, apex truncate; dorsoventrally convex, apex not visible in dorsal view; disc with longitudinal lines of coarse punctures, and indistinct longitudinal grooves along punctures present only near base, no ridges present between longitudinal grooves. Gonocoxae (Fig. 26F) slender, connected from basal 1/5 to base; each gonocoxa with seven long and one tiny setae from apical 1/5 to apex, subapically slightly curved. Ventrite VIII (Fig. 26E) weakly sclerotized apically, with several short setae at sides of apex, spiculum extremely elongate. Spermathecal receptaculum (Fig. 26G) strongly swollen, with transverse wrinkles at basal 1/2; pump wide and curved, with transverse wrinkles at apical 2/3; sclerotized spermathecal canal moderately long before base of spermathecal gland.

#### Food plants.

Rosaceae: *Rubus corchorifolius* L. f.

#### Etymology.

This new species is named for Su-Fang Yu (余素芳), the first member of TCRT to collect specimens.

#### Distribution.

This species is widespread at lowlands of northern Taiwan (Fig. 13).

### ﻿Key to Taiwanese species of *Batophila*

**Table:** 

1	Elytra abruptly, apically narrowed (Figs 5, 21)	**2**
–	Elytra gradually, apically narrowed (Figs 2, 10, 12, 16, 17, 19, 24)	**4**
2	Elytra dorsoventrally convex, apex not visible in dorsal view in both sexes; aedeagus parallel-sided (Fig. 5C, F)	***B. choui* sp. nov.**
–	Elytra dorsoventrally convex only in female but flattened in males; aedeagus subapically widened (Fig. 12C, F)	**3**
3	Aedeagus slightly wide in apical 1/5 (Fig. 22C)	***B. tsoui* sp. nov.**
–	Aedeagus extremely wide in apical 1/5 (Fig. 8C)	***B. chungi* sp. nov.**
4	Elytral apices convergent, or divergent and rounded (Figs 17, 19, 24)	**5**
–	Elytral apices truncate (Figs 2, 10, 12, 16)	**7**
5	Antennae stout, antennomeres VI–X 0.5 as long as antennomere I, apex of aedeagus widely rounded (Figs 24, 25A, B)	***B. yehi* sp. nov.**
–	Antennae slender, antennomeres VI–X > 0.5 as long as antennomere I (Figs 18A, B, 20A, B)	**6**
6	Longitudinal ridges on elytra distinct and sexually dimorphic; apex of aedeagus truncate (Fig. 18A, B)	***B. meihuai* sp. nov.**
–	Longitudinal ridges on elytra instinct or reduced; apex of aedeagus rounded but with one rounded process at middle (Fig. 20A, B)	***B. taiwanica* Döberl**
7	Punctures on elytra tiny (Fig. 2); aedeagus subapically widened (Fig. 3C)	***B. alishanensis* sp. nov.**
–	Punctures on elytra coarse (Figs 10, 12, 16); aedeagus apically narrowed or parallel sided	**8**
8	Apex of aedeagus tapering (Figs 15C, 23C)	**9**
–	Apex of aedeagus rounded, but with one process at middle of apical margin (Figs 11C, 14C, 26C)	**10**
9	Apex of aedeagus tapering from apical 1/10; in high mountains of Taichung and Miaoli counties (Fig. 15C)	***B. jungchani* sp. nov.**
–	Apex of aedeagus tapering from apical 1/5; in lowlands of Nantou County (Fig. 23C)	***B. wusheensis* sp. nov.**
10	Apex of aedeagus widely rounded (Fig. 11C); in high mountains of Chiayi, Ilan, Hualien, and Nantou counties	***B. houjayi* sp. nov.**
–	Apex of aedeagus rounded, with one process at middle of apical margin (Figs 14C, 26C)	**11**
11	Rounded apex of aedeagus with one rounded process at middle of apical margin (Fig. 14C); in lowlands of Miaoli County and high mountains of Hsinchu and Taoyuan counties	***B. huangi* sp. nov.**
–	Rounded apex of aedeagus with one truncate process at middle of apical margin (Fig. 26C); in lowlands of Taipei, New Taipei Cities, and Ilan County	***B. yuae* sp. nov.**

## ﻿Discussion

Species diversity of *Batophila* in Taiwan is unexpectedly high by comparison with ten species in mainland China (Bezděk and Konstantinov 2024), probably because taxonomic studies have been hampered by the small body sizes. Specimens smaller than 2.0 mm can be damaged during aedeagal dissections. Moreover, diagnostic characters were difficult to observe historically due to the absence of modern optics and insufficient numbers of specimens. Species are adapted to various microhabitats ranging from alpine areas to lowlands, and adults are wingless. Although high species numbers occur in several genera, including *Paraplotes* Laboissière (Lee 2015) *Agetocera* Hope (Lee et al. 2010), *Sikkimia* Duvivier (Lee and Bezděk 2016), *Siemssenius* Weise (Lee 2016), *Shairella* Chûjô (Lee and Beenen 2017), *Lochmaea* Weise (Lee 2018), and *Furusawaia* Chûjô (Lee and Bezděk 2021), only *Paraplotes* have similar high species diversity (> 10 species) as *Batophila*. In addition to allopatric speciation, two species in the same areas also occur in both genera.

At many localities two species of *Batophila* occur sympatrically. Finding more than two species of *Batophila* at some localities was unexpected. These included Alishan (阿里山), Sungkang (松崗), and Tsuifeng (翠峰). Based on recent field collections, specimens collected from a single host plant belonged to one *Batophila*-species. Further investigations are needed to clarify host specificity in different localities.

In this study the aedeagus is found to have more diagnostic value than previously recognized, especially in the position of the dorsal opening and membranous area on the ventral surface. However, diagnostic value of female genitalia is very limited in this genus by comparison with those of *Argopistes* Motschulsky (Lee et al. 2024). Some diagnostic external morphological characters were found, such as shape of elytra and antenna, and punctuation on the elytra. Although some species are not externally distinguishable, they can be identified by their allopatric distributions and aedeagi.

Various species of *Rubus* (Rosaceae) were confirmed as dominant host plants for members of *Batophila* in Taiwan. Species of this genus in Europe are restricted to species of Rosaceae (Jolivet and Hawkeswood 1995). Interestingly, some Taiwanese species feed on plants in other families. For example, adults of *B. houjayi* sp. nov. feed on leaves of *Persicaria thunbergia* and *P. chinense* (Polygonaceae), and those of *B. tsoui* sp. nov. feed on leaves of *Otanthera scaberrima* (Melastomataceae). Thus, species diversity of *Batophila* of the world is likely still underestimated.

## Supplementary Material

XML Treatment for Batophila acutangula

XML Treatment for Batophila alishanensis

XML Treatment for Batophila choui

XML Treatment for Batophila chungi

XML Treatment for Batophila houjayi

XML Treatment for Batophila huangi

XML Treatment for Batophila jungchani

XML Treatment for Batophila meihuai

XML Treatment for Batophila taiwanica

XML Treatment for Batophila tsoui

XML Treatment for Batophila wusheensis

XML Treatment for Batophila yehi

XML Treatment for Batophila yuae

## ﻿Additional information

### Use of AI

No use of AI was reported.

### Author ORCIDs

Chi-Feng Lee https://orcid.org/0000-0003-1996-0557

### Data availability

All of the data that support the findings of this study are available in the main text.
